# Grapevine fatty acid hydroperoxide lyase generates actin-disrupting volatiles and promotes defence-related cell death

**DOI:** 10.1093/jxb/ery133

**Published:** 2018-04-05

**Authors:** Sahar Akaberi, Hao Wang, Patricia Claudel, Michael Riemann, Bettina Hause, Philippe Hugueney, Peter Nick

**Affiliations:** 1 Molecular Cell Biology, Botanical Institute, Karlsruhe Institute of Technology, Fritz-Haber-Weg, Building, Karlsruhe, Germany; 2 Joint research unit for grapevine health and wine quality (SVQV), INRA, Université de Strasbourg, Colmar, France; 3 Cell and Metabolic Biology, Leibniz Institute of Plant Biochemistry (IPB), Weinberg, Halle (Saale), Germany

**Keywords:** Actin, grapevine (*Vitis vinifera*), green leaf volatiles, hydroperoxide lyase, jasmonic acid, programmed cell death

## Abstract

Fatty acid hydroperoxides can generate short-chained volatile aldehydes that may participate in plant defence. A grapevine hydroperoxide lyase (VvHPL1) clustering to the CYP74B class was functionally characterized with respect to a role in defence. In grapevine leaves, transcripts of this gene accumulated rapidly to high abundance in response to wounding. Cellular functions of *VvHPL1* were investigated upon heterologous expression in tobacco BY-2 cells. A C-terminal green fluorescent protein (GFP) fusion of VvHPL1 was located in plastids. The overexpression lines were found to respond to salinity stress or the bacterial elicitor harpin by increasing cell death. This signal-dependent mortality response was mitigated either by addition of exogenous jasmonic acid or by treatment with diphenyleneiodonium (DPI), an inhibitor of NADPH oxidases. By feeding different substrates to recombinantly expressed enzyme, VvHPL1 could also be functionally classified as true 13-HPL. The cognate products generated by this 13-HPL were *cis*-3-hexenal and *trans*-2-hexenal. Using a GFP-tagged actin marker line, one of these isomeric products, *cis*-3-hexenal, was found specifically to elicit a rapid disintegration of actin filaments. This response was not only observed in the heterologous system (tobacco BY-2), but also in a grapevine cell strain expressing this marker, as well as in leaf discs from an actin marker grape used as a homologous system. These results are discussed in the context of a role for *VvHPL1* in a lipoxygenase-dependent signalling pathway triggering cell death-related defence that bifurcates from jasmonate-dependent basal immunity.

## Teaser

A fatty acid hydroperoxide lyase from grapevine promotes defence-related cell death and generates 3-*cis*-hexenal, which specifically activates actin disruption.

## Introduction

Plant immunity relies, to a large degree, on inducible defence. The plant response to pathogens is complex and composed of at least two layers: a basal, broad-band immunity [pathogen-associated molecular pattern (PAMP)-triggered immunity, PTI] co-exists with a strain-specific level of immunity (effector-triggered immunity, ETI), which often culminates in a specific type of programmed cell death (PCD) termed hypersensitive response (HR) and originates from a co-evolutionary history between host and pathogen (Jones and Dangl, 2006).

The qualitatively different output of PTI and ETI would suggest that the underlying signalling must be different. However, the model of a strict difference is frequently questioned (reviewed in Thomma *et al.*, 2011). In fact, a comparative study in grapevine cells (Chang and Nick, 2012) has revealed that the early signals for PTI and ETI are mostly identical, but are generated in a different temporal pattern.

One of these generic signals are reactive oxygen species (ROS). Originally just seen as a manifestation of impaired redox balance, they are now recognized as central signals, whereby specificity comes from their temporal and spatial patterns (reviewed in Miller *et al.*, 2010; Ismail *et al.*, 2014). In plants, specific members of the NADPH oxidases (respiratory burst oxidase homologues, RboHs) play a key role in the signal-related production of ROS (reviewed in Marino *et al.*, 2012). Located in the cell membrane, these NADPH oxidases can transfer electrons to O^2^ and generate the superoxide anion, which is later converted to H_2_O_2_ as a further signal used for transduction. In grapevine cells, RboH was shown to regulate defence-related cell death through modulating actin organization (Chang *et al.*, 2015), or to activate innate immunity by activation of the transcription factor gene *MYB14* that up-regulates stilbene synthase, a key enzyme of phytoalexin synthesis (Duan *et al.*, 2016).

Membrane lipids represent an important target of ROS. While non-enzymatic lipoxygenation is considered as a stress damage event (reviewed in Farmer and Mueller, 2013), enzymatic lipoxygenation is beneficial, because it activates stress adaptation. Lipoxygenase generates hydroperoxy fatty acids including 13-hydroperoxides (13-HPOD/T) and 9-hydroperoxides (9-HPOD/T) that are subsequently converted by enzymes belonging to the CYP74 clade of the cytochrome P_450_ superfamily. This clade differs from canonical cytochrome P_450_ proteins, because its members need neither oxygen nor NADPH oxidoreductase as cofactor for their activity, and also show low affinity for carbon monoxide. The CYP74 clade is subdivided into different branches: allene oxide synthase (AOS; CYP74A) as the first committed step of jasmonic acid (JA) biosynthesis, and canonical hydroperoxide lyase (HPL; CYP74B) leading to the formation of aldehydes. Both enzymes compete for the same substrate, 13-hydroperoxy octadecatrienoic acid (13-HPOT), and act antagonistically. In fact, down-regulation of the HPL branch has been shown to activate jasmonate synthesis (Liu *et al.*, 2012; Tong *et al.*, 2012). In contrast to CYP74A and CYP74B, the CYP74C-type HPL accepts both 13- and 9-hydroperoxides. Both CYP74B- and CYP74C-type HPLs are responsible for the emission of small aldehydes that act as green leaf volatiles (GLVs). The CYP74 subclades might have arisen from gene duplications followed by divergent mutations in the active centre by exchange of individual amino acids, since it is possible to convert an AOS into a HPL (Lee *et al.*, 2008).

Although the HPL branch competes with the AOS branch, it is also involved in plant defence. The volatile products of HPL have received considerable attention for their role in the response to herbivory (reviewed in Gershenzon, 2007), but there is evidence that they can also act as signals to activate systemic defence (Farag *et al.*, 2005; Frost *et al.*, 2008).

**Fig. 1.**
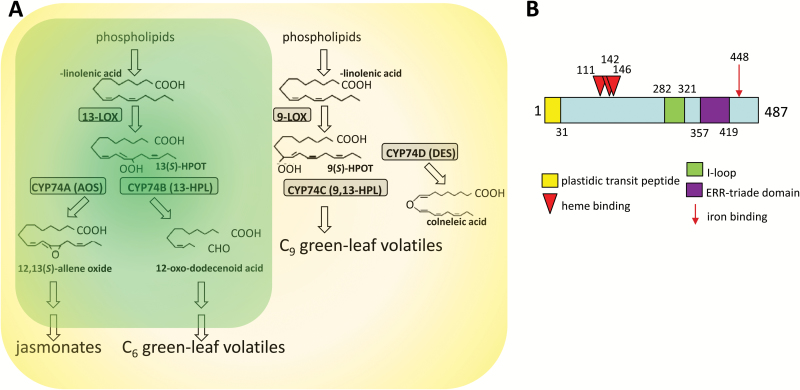
Molecular features and gene ontology of the HPL1 isolated from *Vitis vinifera* cv. ‘Müller-Thurgau’ in the context of the CYP74 family. (A) Simplified scheme for the metabolic pathways driven by the different subclades according to Hughes *et al.* (2009). LOX, lipoxygenase; HPOT, hydroperoxy octadecatrienoic acid; AOS, allene oxide synthase; HPL, hydroperoxide lyase; DES, divinyl ether synthase. Plastidic localization is indicated by green shading in the case of 13-HPLs. In contrast, many 9/13 HPL (CYP74C) are extraplastidial (yellow zone). A molecular phylogeny of the CYP74 family is given in Supplementary Fig. S1. A full alignment of the HPL isolated from *V. vinifera* cv. ‘Müller-Thurgau’ along with representatives of the different CYP74 subclades and the subclade-specific signatures is given in Supplementary Fig. S2. (B) Molecular features of the HPL isolated from *Vitis vinifera* cv. ‘Müller-Thurgau’ according to Toporkova *et al.* (2013). Substrate binding is located in the I-loop (corresponding to the oxygen-binding domain in other cytochrome P_450_ proteins); the ERR triad domain is characteristic for the CYP74 family and modulates substrate specificity.

The 13-HPLs (CYP74B) can catalyse the cleavage of 13-HPOD/T derived from linoleic acid or linoleneic acid, leading to the production of C_6_ GLVs (Matsui, 2006). The aldehydes are later reduced and converted to C_6_ alcohols and esters by the action of alcohol dehydrogenase and isomerase (Bate and Rothstein, 1998). C_6_ volatiles are considered to be involved directly in plant defence as antimicrobials. For instance, *trans*-2-hexenal exerts antibacterial (Croft *et al.*, 1993) as well as antifungal activity (Hamilton-Kemp *et al.*, 1992). However, C_6_ volatiles can also act as signalling molecules and induce defence genes (Bate and Rothstein, 1998; Scala *et al.*, 2013) or give rise to the wound hormone traumatin involved in wound healing (Siedow, 1991). In contrast to the CYP74B HPLs, the 9-/13-HPLs (CYP74C) accept both (13-HPOD/T) and (9-HPOD/T) hydroperoxy fatty acids, generating both C_6_ and C_9_ GLVs (Fig. 1A). Also C_9_ volatiles are involved in defence responses against pathogens and have been shown to impair hyphal growth (Hamilton-Kemp *et al.*, 1992).

The fact that HPL forms differing in their expression patterns generate distinct patterns of volatile aldehydes (Chehab *et al.*, 2006) suggests that the chemical complexity generated by these enzymes is of biological relevance. It is also clear that their role in defence is distinct from that of the AOS-derived jasmonates (Chehab *et al.*, 2008), although both branches interact. For instance, silencing HPL and AOS enzymes in *Nicotiana attenuata* plants enhanced GLV and JA levels in response to herbivores (Halitschke *et al.*, 2004).

In our previous work, we had mapped defence responses in cell cultures from grapevine. In this system, PTI can be induced by a 22 amino acid peptide (the PAMP flg22) derived from a conserved flagellin domain, whereas the bacterial elicitor harpin induced an ETI-like immunity culminating in cell death (Chang and Nick, 2012). In the same system, we recently could show that flg22 induced the accumulation of JA and its isoleucine conjugate JA-Ile, while harpin did not. This assigned the activation of jasmonate synthesis clearly to basal, but not to cell death-related defence (Chang *et al.*, 2017) leading to the question of whether the bifurcation of the oxylipin pathway produced by the duplication into the CYP74A (AOS) jasmonate-generating branch and the CYP74B (HPL) volatile-generating branch might be linked with the bifurcation of defence signalling that leads either to basal immunity (AOS, jasmonate pathway) or to a cell death-related immunity (HPL, volatile pathway). So far, two HPL genes (*VvHPL1* and *VvHPL2*) had been isolated from *Vitis vinifera* L. ‘Cabernet Sauvignon’ berries and were characterized with respect to their molecular properties (Zhu *et al.*, 2012), but the function of grapevine HPL in defence responses has not been addressed. Therefore, we overexpressed a 13-HPL isolated from the cultivar Müller-Thurgau in the tobacco cell line BY-2, and studied its subcellular localization and phenotype. By feeding 9-HPOT and 13-HPOT to the recombinantly expressed HPL, we could show that this HPL accepts only 13-HPOT as substrate and generates *cis*-3-hexenal along with *trans*-2-hexenal. We show further that only *cis*-3-hexenal evokes a specific actin response, which is followed by rapid cell death. This actin response can be demonstrated both in tobacco BY-2 and in suspension cells of grapevine itself. In leaf epidermis of grapevine, it is also present but here the isomeric 2-*trans*-hexenal, which is inactive in isolated cells, can also trigger a response of actin. We arrive at a model where HPL channels the defence-related oxidative burst towards cell death-related immunity, using *cis*-3-hexenal as a signal.

## Materials and methods

### Cloning of *VvHPL1*

Leaves of *V. vinifera* cv. ‘Müller-Thurgau’ were collected from plants in the greenhouse of the Karlsruhe Institute of Technology, and immediately frozen in liquid nitrogen. Frozen tissues (50–70 mg) were ground prior to extraction of total RNA using a Spectrum™ Plant Total RNA Kit (Sigma-Aldrich, Deisenhofen, Germany). For cDNA synthesis, 1 μg of RNA was subjected to reverse transcription as described in Duan *et al.* (2016), based on the published sequence (Zhu *et al.*, 2012; accession HM627632) using the oligonucleotide primers shown in Supplementary Table S1 at *JXB* online. The sequence of the amplicon (accession KX379687) was verified by sequencing and then it was inserted into the binary vector pH7FWG2,0 (Karimi *et al.*, 2002), using Gateway technology (Invitrogen Corporation, Paisley, UK), pH7WGF2 [N-terminal fusion of green fluorescent protein (GFP)], and pH7FWG2 (C-terminal fusion of GFP).

### Cell lines

Cells of the strain *Nicotiana tabacum* L. cv. ‘Bright Yellow 2’ (BY-2; Nagata *et al.*, 1992) were cultivated as described in Rajabi *et al.* (2017). To visualize actin filaments *in vivo*, a transgenic tobacco BY-2 line expressing the actin-binding domain of plant fimbrin (FABD2) in fusion with GFP was used (Sano *et al.*, 2005). To visualize actin filaments in grapevine leaves, a transgenic grape (*V. vinifera* L. cv. ‘Chardonnay’) expressing the FABD2–GFP marker was used, as well as a suspension cell culture derived from regenerating calli of the same genotype (Guan *et al.*, 2014) that were cultivated as described in Wang and Nick (2017). Transgenic suspension cells were subcultivated in Murashige and Skoog (MS) medium complemented with the respective antibiotics (45 mg l^–1^ hygromycin in the case of the transgenic BY-2 lines, 30 mg l^–^ kanamycin in the case of the *Vitis* cells).

### Transformation of tobacco BY-2 cells

A BY-2 cell line overexpressing VvHPL1–GFP in a stable manner was generated according to Buschmann *et al.* (2011) with some modifications according to Gao *et al.* (2016) using chemo-competent *Agrobacterium tumefaciens* (strain EHA105) for the transformation.

### Stress and inhibitor treatments

All the compounds tested were added into the medium at the time of subcultivation. As abiotic stressor, NaCl was administered, to activate basal defence; flagellin fragment flg22 (antikoerper, Aachen, Germany), dissolved in sterile water, was given at 1 µM. To activate cell death-related defence, harpin (Pflanzenhilfsmittel, ProAct, Starnberg, Germany) was used at a concentration of either 18 µg ml^–1^ or 27 µg ml^–1^. In some experiments, cells were treated with 100 μM (±)-JA (Sigma-Aldrich, Germany), or with 200 nM of the inhibitor of NADPH oxidase, diphenyleneiodonium (DPI) (Cayman, USA).

### Microscopical analysis of the cell lines

Fluorescent proteins were observed using the AxioObserver Z1 (Zeiss, Jena, Germany) inverted microscope equipped with a laser dual spinning disc scan head from Yokogawa (Yokogawa CSU-X1 Spinning Disk Unit, Yokogawa Electric Corporation, Tokyo, Japan), a cooled digital CCD camera (AxioCamMRm; Zeiss), and two laser lines (488 nm and 561 nm, Zeiss, Jena, Germany) attached to the spinning disc confocal scan head. Images were recorded using a Plan-Apochromat ×63/1.44 DIC oil objective operated via the Zen 2012 (Blue edition) software platform. To test for a potential co-localization of the fusion protein with plastids, the tpFNR-mEosFP (Schattat *et al.*, 2012; a kind gift of Professor Dr Jaideep Mathur, Guelph University, Canada) was transiently transformed into the VvHPL1–GFP overexpressors using the *Agrobacterium*-based protocol (Buschmann *et al.*, 2011) described above. Mortality was determined using the Evans Blue dye exclusion test (Gaff and Okong’O-Ogola, 1971) as described in Kühn *et al.* (2013). Mitotic indices were followed over time after staining with Hoechst 22358 (Sigma-Aldrich, Neu-Ulm, Germany), and cell width and length were quantified using the MosaiX module of the imaging software (Axiovision, Zeiss, Jena, Germany) as described in Kühn *et al.* (2013).

### Measuring expression of HPL1–GFP

To verify overexpression of the VvHPL1–GFP fusion protein, cells from the BY-2 and the HPL1-overexpressing (HPL1ox) line were collected at day 3 after subcultivation, and extracts of soluble and microsomal proteins were obtained according to Jovanović *et al.* (2010), and analysed by SDS–PAGE and western blotting according to Nick *et al.* (1995). After removing the medium by centrifugation for 10 min at 4 °C at 13 000 *g* (Heraeus Pico 17 Centrifuge, 600 Thermo Scientific, Langenselbold, Germany), cells were homogenized according to Nick *et al.* (1995), with some modifications, in the same volume of extraction buffer containing 25 mM MES, 5 mM EGTA, 5 mM MgCl_2_, pH 6.9, supplemented with 1 mM DTT, and 1 mM phenylmethylsulphonyl fluoride (PMSF) by using a glass potter with a narrow gap for 15 min on ice. Cell lysates were first spun down at 13 000 *g* for 5 min to remove nuclei and debris. The supernatant was then ultracentrifuged at 100 000 *g*, 4 °C for 15 min (rotor TLA 100.2, Beckman, Munich, Germany) to yield a soluble fraction (containing cytosolic proteins) and a microsomal fraction (containing plasma membrane, endomembrane, mitochondrial, and plastidic proteins). The fusion of VvHPL1 was detected using antibodies against the GFP reporter (Sigma-Aldrich) at a dilution of 1:1000. For signal development, a goat polyclonal anti-mouse IgG conjugated to alkaline phosphatase (Sigma-Aldrich) in a dilution of 1:2500 was employed. Equal loading was verified by running a replicate gel that was stained with Coomassie Brilliant Blue.

### Recombinant expression of *VvHPL1*

The coding sequence of the VvHPL1 was inserted into the pET-DEST42 Gateway vector. The resulting construct encoding the HPL1 protein fused with a 6× His-tag was expressed in *Escherichia coli* (ER2566). The entire volume of a 60 ml pre-culture was inoculated into 3 litres of LB medium supplemented with 100 mg l^–1^ ampicillin and cultivated at 37 °C. When an OD_600_ of 0.8–1.0 had been reached, 500 µM isopropyl-β-d-thiogalactopyranoside (IPTG) was added. After incubation (18 °C, 20 h), cells were harvested by centrifugation (10 000 *g*, 20 min, 4 °C), and proteins were extracted according to Mu *et al.* (2012) with minor modifications: cells were washed with 50 mM sodium phosphate buffer (pH 7.0), spun down again, and suspended in 20 ml of 50 mM Tris–HCl, 500 mM NaCl, 0.5% Triton X-100, and 0.5 mM PMSF (pH 8.0), and lysed twice (French Press, 1000 bar). Cell debris was removed by centrifugation (15 000 *g*, 30 min, 4 °C), and the supernatant fraction was applied onto Ni-NTA agarose to purify the fusion protein. The concentrated eluates were then separated by SDS–PAGE on 10% acrylamide gels and the fusion protein was detected by western blot using a monoclonal mouse anti-histidine antibody 1:2000 (Penta.His Antibody, BSA-free, Qiagen) diluted in Tris-buffered saline.

### SBSE analysis of HPL products

For analysis by stir bar sorptive extraction (SBSE), a stir bar (0.5 mm film thickness, 10 mm length, Gerstel GmbH, Mülheim an der Ruhr, Germany) coated with polydimethylsiloxane fibres (PDMS; Supelco, Bellefonte, PA, USA) was used. The stir bar was placed in a 20 ml glass headspace vial along with the reaction medium [2 ml containing 20 µg of partially purified recombinant VvHPL1, 2 ml of 50 mM sodium phosphate buffer (pH 7.0), and 40 µM of either 13(S)-hydroperoxy-9(Z), 11(E)-octadecadienoic acid (HPOD; Larodan, Sweden) or 13(S)-hydroperoxy-9(Z),11(E), 15(Z)-octadecatrienoic acid (HPOT; Larodan, Sweden)], and 2 ml of a 5 M NaCl solution. The SBSE (simultaneous incubation and extraction) was performed at ambient temperature while stirring (2 h, 250 rpm). After extraction, the stir bar was transferred into a glass desorption tube and was thermally desorbed. The desorbed compounds were analysed by thermal desorption–GC-MS (TD-GC-MS).

The TD-GC-MS analysis was performed with a thermal desorption unit (TDU) equipped with an MPS2 auto-sampler and a cryostatic cooled CIS-4 programmed temperature vaporization (PTV) inlet (Gerstel) installed on an Agilent 7890B gas chromatograph with a 5977B mass-selective detector (Agilent Technologies).

The stir bars were thermally desorbed by programming the TDU from 30 °C (held for 1 min) to 250 °C (held for 5 min) at 60 °C min^–1^ with 70 ml min^–1^ desorption flow. Desorbed compounds were focused at –20 °C on a baffled glass liner in the cooled PTV inlet for subsequent GC-MS analysis. After desorption, the PTV inlet was programmed from –20 °C to 300 °C (held for the GC run time) at 10°C s^–1^ to inject trapped compounds onto the analytical column. The injection was performed in the solvent vent mode with a split vent flow of 20 ml min^–1^.

A HP-5MS capillary column (30 m, 0.25 mm id, 0.25 µm film thickness, Agilent Technologies) was used for separation. The chromatographic program was set at 40 °C for 10 min, raised to 300 °C (at 4 °C min^–1^), and held at the final temperature for 10 min. A constant helium flow of 1 ml min^–1^ was used. The temperatures for the MS transfer line and ion source were 270 °C and 230 °C, respectively. Ionization voltage was 70 eV. Full scan mode was used for acquiring the data from *m/z* 30 to 450. All data were recorded using MS ChemStation.

Detected volatiles were identified by comparing mass spectra with those of authentic standards and spectral libraries. The NIST-14 mass spectral libraries were used for identification. Compounds were considered to be positively identified after matching of both mass spectra and linear retention indices (LRIs) with those of authentic samples. The LRI was calculated from a compound retention time relative to the retention of a series of *n*-alkanes (C_8_–C_28_).

### Quantification of JA-Ile content

For hormonal analysis, non-transformed BY-2 wild-type as well as HPL-overexpressing cells were harvested after treatment with 27 µg ml^–1^ harpin for either 30 min or 3 h, respectively, or with an equal volume of water as solvent control. The culture medium was removed by vacuum using a Büchner funnel, the fresh weight was determined, and the drained cells were frozen in liquid nitrogen. Contents of the isoleucine conjugate of jasmonic acid (JA-Ile) were quantified using a standardized ultraperformance LC-MS (UPLC-MS/MS)-based method and [^2^H_2_]JA-Ile as internal standard according to Balcke *et al.* (2012).

### Actin response to C_6_ volatiles

The cellular effects of VvHPL1 products were examined in different systems expressing a GFP fusion of the actin-binding domain 2 of *Ath* fimbrin 1. Both *cis*-3-hexenal (50% purified in triacetine, Sigma-Aldrich) and *trans*-2-hexenal (98% purified, Sigma-Aldrich) were tested for their putative role in signalling and defence responses. In the case of transgenic tobacco BY-2 (Sano *et al.*, 2005) and transgenic *V. vinifera* cv. ‘Chardonnay’ (Guan *et al.*, 2014), cells were collected at day 3 after subcultivation on 0.8% MS agar, and 1 µl of *cis*-3-hexenal, *trans*-2-hexenal, or the solvent triacetin/water was placed on 10 mm sterile filter paper discs in the centre of the MS agar plate and incubated for 10 min. After incubation, cells were transferred to MS medium. Cells were then followed over time by spinning disc microscopy as described above. In the case of transgenic grapevine plants (Guan *et al.*, 2014), fully expanded leaves (leaf 4 from the top) were used in two orientations to view both the abaxial and the adaxial side of the leaf.

### Quantification of steady-state levels of VvHPL1 transcripts

To follow the expression of *HPL1* in grapevine in response to wounding, the detached fourth leaf from the shoot apex of *V. vinifera* cv. ‘Müller-Thurgau’ plants from the greenhouse of the Botanical Garden of the Karlsruhe Institute of Technology were wounded 20 times using a 5 mm cork borer to simulate herbivore damage. Leaves were frozen at 2, 4, and 6 h after the treatment along with the non-wounded control in liquid nitrogen and kept in a –80 °C freezer until RNA extraction and reverse transcription conducted as described above. The transcript level of the *VvHPL1* was determined by quantitative real-time PCR (qPCR), using the primers given in Supplementary Table S3. As internal standard, the ubiquitin conjugating enzyme (UBC) was selected (Reid *et al.*, 2006), and qPCR analysis was carried out according to Gutjahr *et al.* (2008) in three technical replicates from each of the three biological replicates. Relative expression levels of *VvHPL1* over UBC were calculated with the delta delta C_t_ method (Livak and Schmittgen, 2001) using UBC as the endogenous control for normalization. The experiment was repeated for three biological replicates, and the mean fold change was calculated and plotted along with corresponding standard errors. This protocol was adapted from Svyatyna *et al.* (2014). The steady-state levels of overexpressed *VvHPL1* in the transgenic tobacco BY-2 cells were measured by the same approach.

## Results

### HPL1 from *Vitis vinifera* shows all the features of a canonical CYP74B

A HPL1 cDNA of the predicted size (1464 bp) was amplified from *V. vinifera* cv. ‘Müller-Thurgau’ leaves, which is predicted to encode a polypeptide of 487 amino acids with a calculated molecular mass of 55 kDa. The predicted protein sequence was aligned with other CYP74 sequences obtained from Swissprot (www.expasy.org) using the ClustalW algorithm. A Neighbor–Joining tree was constructed and subjected to a bootstrap test based on 500 replicates (Supplementary Fig. S1). The VvMTh-HPL1 protein is identical to a protein which has been isolated from the *V. vinifera* cultivar ‘Cabernet Sauvignon’, except for one residue, and shown experimentally to be a canonical CYP74B accepting 13-HPOD/T as substrates (Zhu *et al.*, 2012). Other members of this clade were also experimentally confirmed as 13-HPLs (Supplementary Fig. S1). Moreover, the VvMTh-HPL1 sequence displays all molecular features characteristic of a canonical CYP74B (Fig. 1B): (i) an N-terminal plastid transit peptide comprising amino acid residues 1–31 as predicted by two algorithms, targetP (http://www.cbs.dtu.dk/services/TargetP/) and ChloroP (http://www.cbs.dtu.dk/services/ChloroP/); (ii) a cysteine triade signature relevant for haem binding at positions 111, 142, and 146; (iii) a substrate-binding I-loop; (iv) the ERR triad domain characteristic for the CYP74 family; and (v) a highly conserved iron-binding cysteine at residue 438. The different subclades of the CYP74 family have specific molecular features related to substrate specificity (Supplementary Fig. S2). Based on these features, the VvMTh-HPL1 clearly qualifies as a canonical member of the CYP74B clade. Specifically, VvMTh-HPL1 harbours a leucine in position 115 instead of a phenylalanine in grapevine AOS. The mutation of this crucial leucine to phenylalanine has been shown to convert HPL activity into AOS activity (Lee *et al.*, 2008). Likewise, a second feature of this HPL signature, an alanine in position 133 (contrasting with a serine in AOS), is also present, showing that VvMTh-HPL1 displays the molecular signature for a 13-HPL (Lee *et al.*, 2008).

### HPL1 from *Vitis vinifera* is localized in the plastidic stroma

**Fig. 2.**
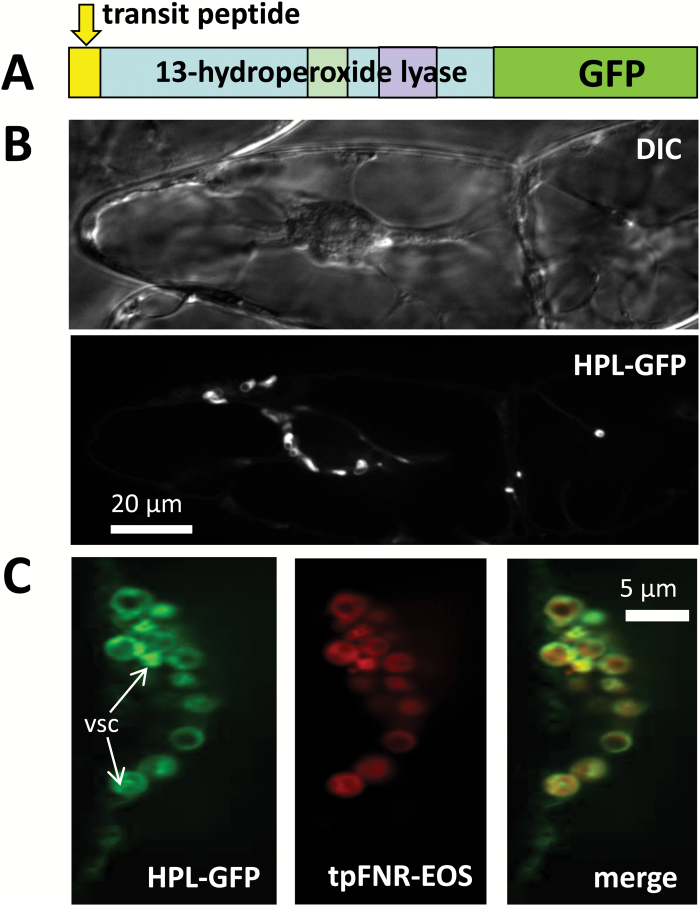
Localization of the HPL1 isolated from *Vitis vinifera* cv. ‘Müller-Thurgau’ overexpressed in tobacco BY-2 cells. (A) Structure of the introduced fusion construct of HPL1 with GFP fused to the C-terminus. (B) Representative confocal section from a *z*-stack along with a differential interference contrast (DIC) image of the same cell for the HPL1–GFP fusion. (C) Duplex visualization of GFP fused to the C-terminus of HPL and the stromal marker tpFNR-mEOS. White arrows indicate vesicular structures (vsc) in the proplastid interior, where the HPL–GFP signal accumulates.

As a member of the CYP74B clade, VvHPL1 from ‘Müller-Thurgau’ is expected to localize in the plastids. The presence of a bona fide signal peptide is consistent with this prediction. To verify the subcellular localization of the VvHPL1, a C-terminal fusion of VvHPL1 and GFP was expressed under control of the *35S* promoter in BY-2 cells (Fig. 2A). It appeared in small ovoid organelles (Fig. 2B). Co-expression with the N-terminal fragment of ferredoxin NADPH oxidoreductase including the transit peptide, tpFNR-mEosFP, a stromal marker (Schattat *et al.*, 2012), revealed that these VvHPL1-containing organelles are indeed proplastids.

### Recombinant VvHPL1 prefers 13-hydroperoxy-fatty acids as substrate

**Fig. 3.**
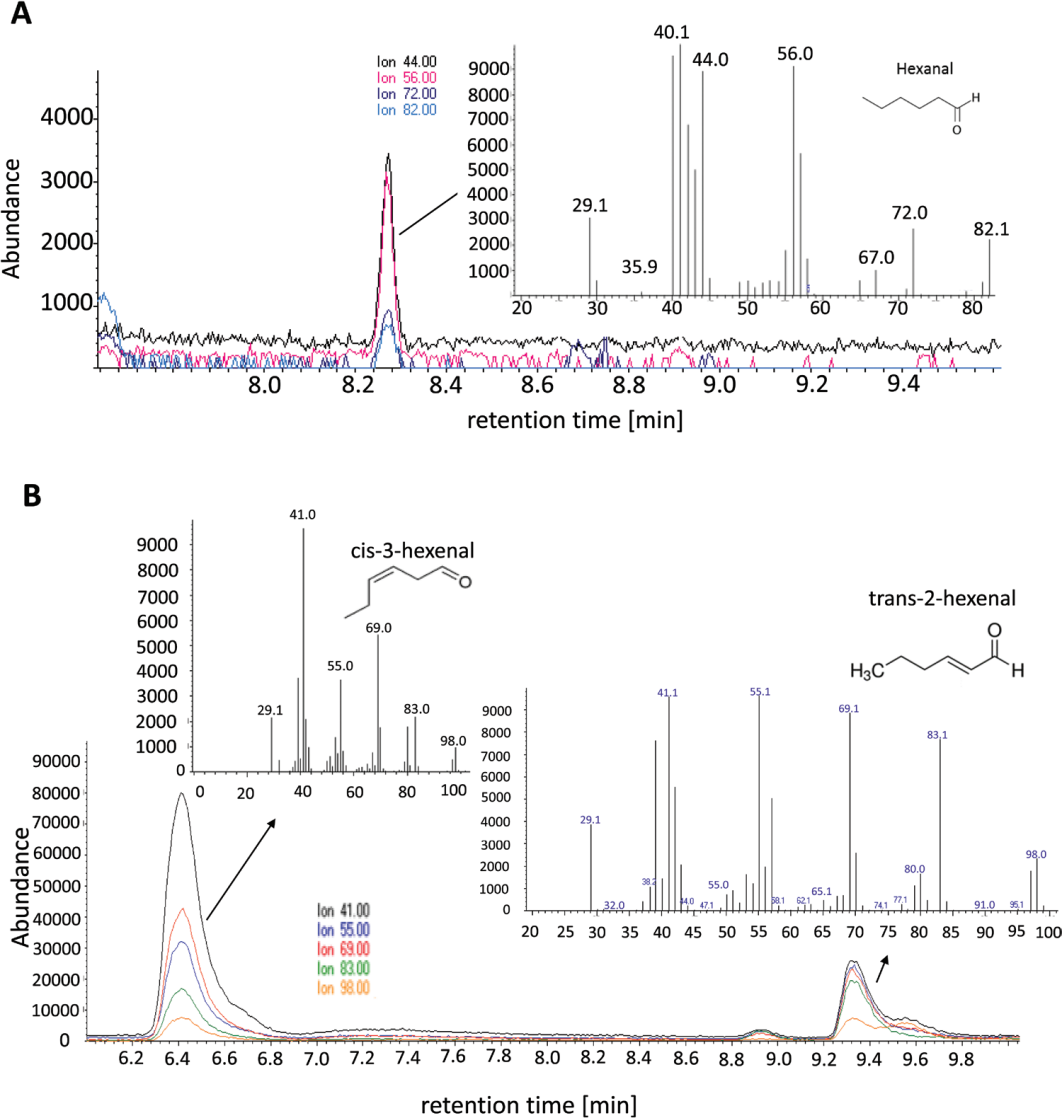
VvHPL1 substrate specificity assay performed with the recombinant enzyme. Ion chromatograms of the extracts along with mass spectra of the prominent peaks for feeding with 13-HPOD (A) and with 13-HPOT (B). The mass spectra of the indicated peaks match those for hexanal (A), and *cis*-3-hexenal and *trans*-2-hexenal (B), respectively.

To characterize the substrate specificity, the full-length coding sequence of *VvHPL1* was cloned into the pET-DEST42 vector for recombinant expression. The recombinant VvHPL1 protein was soluble and showed the predicted mol. wt of 59 kDa. The purified recombinant VvHPL1 was incubated with all four potential hydroperoxy-fatty acid substrates (13-HPOD, 13-HPOT, 9-HPOD, and 9-HPOT). The recombinant VvHPL1 was able to catalyse the cleavage of either 13-HPOD or 13-HPOT into C_6_ volatiles (Supplementary Table S2), but did not show any activity towards 9-HPOD or 9-HPOT. The major product from the conversion of 13-HPOD was hexanal (Fig. 3A), while 13-HPOT as substrate yielded *cis*-3-hexenal and *trans*-2-hexenal (Fig. 3B). These compounds were verified by comparing mass spectra with those of authentic standards and spectral libraries. We could not detect any C_9_ volatiles such as nonanal, 2-nonenal, or (z,z)-3,6-nonadienal, either with 9-HPOD or with 9-HPOT. Moreover, no volatile compounds were detected in control samples using substrates and sodium phosphate buffer in the absence of recombinant protein. Based on these results, the *VvHPL1* gene isolated from ‘Müller-Thurgau’ is concluded to encode a functional 13-HPL, which is able to catalyse the cleavage of 13-hydroperoxy-fatty acids into C_6_-aldehydes and therefore can also functionally be categorized as a member of the CYP74B subfamily.

### The HPL1–GFP-overexpressing cells are more sensitive to salinity

**Fig. 4.**
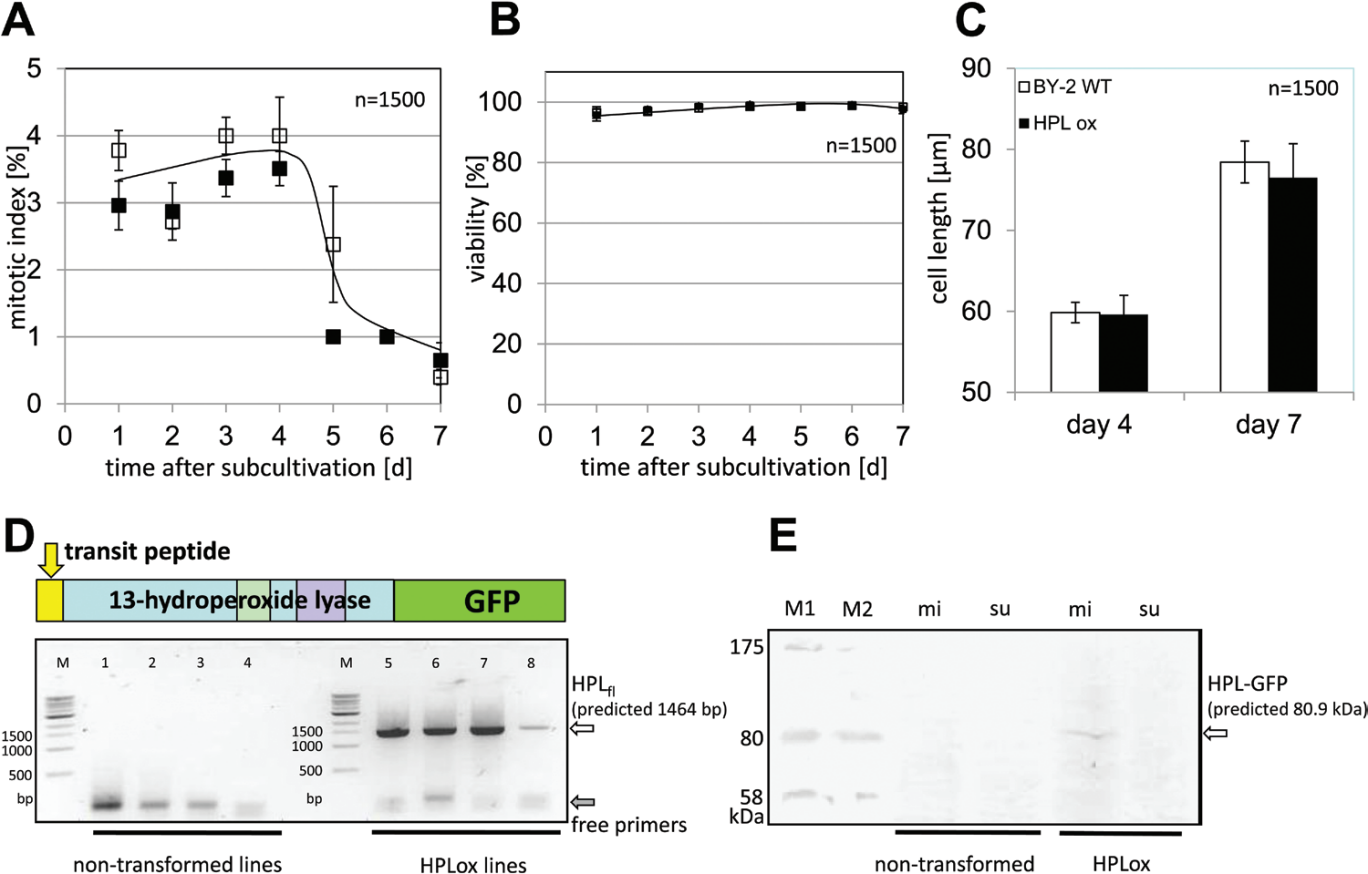
Normal behaviour of tobacco BY-2 cells overexpressing the HPL1 isolated from *Vitis vinifera* cv. ‘Müller-Thurgau’ in fusion with GFP at the C-terminus under physiological conditions. (A–C) Physiological parameters of the HPL1–GFP overexpressor (black) compared with non-transformed BY-2 (white): viability (A) and mitotic index (B) over the cultivation cycle, along with cell length (C) at the end of proliferation phase (day 4) and the end of the expansion phase (day 7). Values represent the mean and SEs from a population of 500 cells per measurement. Differences are not significant using Student’s *t*-test. (D) Steady-state levels of the HPL1 transcript in four samples from non-transformed BY-2 (left) and in four samples from HPL-overexpressing lines (right) probed by RT-PCR with primers spanning full-length HPL1 predicted to produce an amplificate of 1464 bp in length. White arrow, putative full-length HPL1 transcript; grey arrow, free primer pairs. (E) The putative HPL1–GFP fusion protein is exclusively detected by western blotting (using a monoclonal antibody against the GFP tag) in microsomal (mi) fractions from cells overexpressing HPL1–GFP, but not in the cytosolic supernatant of the same cells (su), or in any fraction from non-transformed BY-2 cells. M1 and M2 are two size markers from different commercial products. The estimated size of the putative HPL1–GFP fusion is 80.9 kDa.

**Fig. 5.**
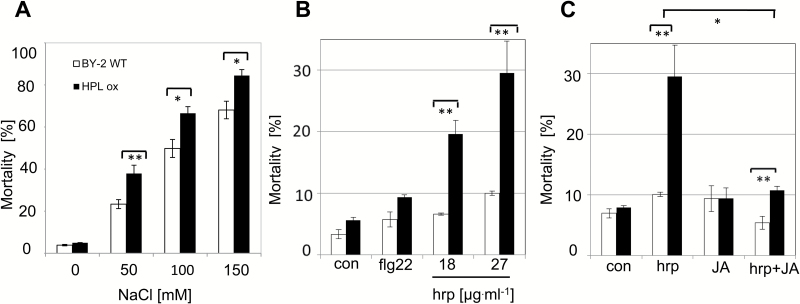
Increased inducibility of signal-dependent cell death in tobacco BY-2 cells overexpressing the HPL isolated from *Vitis vinifera* cv. ‘Müller-Thurgau’ in fusion with GFP at the C-terminus under physiological conditions. (A) Mortality after addition of NaCl in the non-transformed BY-2 (white bars) versus the HPL overexpressor (black bars). (B) Mortality after addition of the PAMP flg22 (10 µM), compared with 18 µg ml^–1^ or 27 µg ml^–1^ harpin. (C) Effect of jasmonic acid (JA, 100 µM) on cell death induced by harpin (27 µg ml^–1^). Mortality was scored after 24 h of treatment. Treatment was initiated 30 min after subcultivation. Values represent the mean and SEs from a population of 500 cells per measurement and at least three independent experimental series. Brackets indicate differences that are significant at *P*=0.05 (*) or *P*=0.01 (**), using a Student’s *t*-test.

In the next step, we asked whether overexpression of HPL1 would cause any phenotypic effects. However, when we followed the phenotype of the HLP1-overexpressing line through a cultivation cycle under normal conditions, we could not detect any deviation from the wild type with respect to viability (Fig. 4A) or mitotic index (Fig. 4B). Even subtle details of physiology, such as cell elongation, were not changed in the overexpressor line (Fig. 4C), although we found high steady-state levels for the *VvHPL1* transcript (Fig. 4D). Moreover, we could also confirm the presence of the HPL1–GFP fusion protein in the microsomal fraction of the transformed cells (Fig. 4E). Thus, although HPL1–GFP is properly expressed, accumulates to easily detectable levels, and is correctly localized in the plastidic stroma, we failed to see any phenotypic effect under normal conditions, indicating that the enzyme is not performing catalysis under resting conditions. We asked, therefore, whether we could uncover a phenotype when we provided the substrate by activation of lipid peroxidation. In suspension cells, lipid peroxidation can be efficiently triggered by salinity (Ismail *et al.*, 2012). HPL1-overexpressing and wild-type BY-2 cells were exposed to different levels of salinity stress, and salt-induced mortality of both cell lines was evaluated 24 h after the treatment (Fig. 5A). Whereas in the control mortality was very low (<5%) in both lines, it increased progressively with increasing salinity. Irrespective of the NaCl concentration, the mortality of HPL1ox cells was significantly higher compared with the wild type. This increase of mortality (~15%) was independent of the salt concentration, but remained constant, although the overall mortality increased strongly from 40% for 50 mM NaCl to ~90% for 150 mM NaCl. The effect of *HPL1* overexpression on mortality was therefore additive.

### HPL1 overexpression increases cell death-related, but not basal defence

We further asked whether we would also find phenotypic differences in the context of defence. In grapevine and BY-2 cells, basal defence can be efficiently triggered by flg22, whereas harpin activates a cell death-related form of defence (Chang and Nick, 2012; Guan *et al.*, 2013). We therefore followed the responses of HPL1ox and the wild type to flg22 and harpin. Mortality was determined 24 h later. While flg22 induced only a slight increase of mortality, the effect of harpin was strong, dose dependent, and specific for the genotype: in the wild type, mortality hardly exceeded 10% (Fig. 5B). In contrast, mortality increased dependent on the concentration of harpin from 19.7% at 18 µg ml^–1^ to 30% at 27 µg ml^–1^ harpin after 24 h in transgenic cells (significantly different at *P*=0.01).

JA is known to activate basal immunity, and we asked whether activation of JA signalling can mitigate the harpin-triggered cell death response observed in the HPL1ox line. Previous experiments had shown that the JA sensitivity of BY-2 is low, such that 100 µM racemic JA is required to activate JA-dependent gene expression (Rajabi *et al.*, 2017). When this concentration of JA was administered prior to harpin treatment, the otherwise strong activation of cell death by harpin could be suppressed (Fig. 5C). In the wild type, this JA pre-treatment could reduce harpin-induced mortality to even lower levels. Thus, exogenous JA acted antagonistically to HPL1 overexpression with respect to the harpin response.

The activation of cell death by harpin treatment is correlated with oxidative burst, which is triggered by the NADPH oxidase located in the plasma membrane. This NADPH oxidase, RboH, can be inhibited by the specific inhibitor DPI (Chang *et al.*, 2011). In fact, pre-treatment of cell cultures for 30 min with 200 nM DPI could significantly reduce the mortality induced by harpin (Supplementary Fig. S3). It should be noted, however, that this rescue of viability remained partial. Nevertheless, these data indicate that the NADPH oxidase participates in the mechanism responsible for the HPL1-dependent stimulation of harpin-triggered cell death.

Since activation of JA signalling mitigated harpin-induced cell death in the HPL1ox line, we wondered whether this antagonism should be mirrored in a transient modulation of the bioactive jasmonate conjugate JA-Ile. In fact, we were able to observe in the wild type a transient reduction of JA-Ile levels by almost 50% at 30 min after treatment with harpin (Supplementary Fig. S4), although 3 h after the treatment the JA-Ile had recovered to its original level. This transient reduction of JA-Ile was not seen in the HPL1ox line. Here, the initial level was maintained (Supplementary Fig. S4), indicating that the overexpression of HPL1 had rendered the jasmonate synthesis pathway insensitive to harpin-triggered signalling.

### *Cis*-3-hexenal specifically disrupts actin

**Fig. 6.**
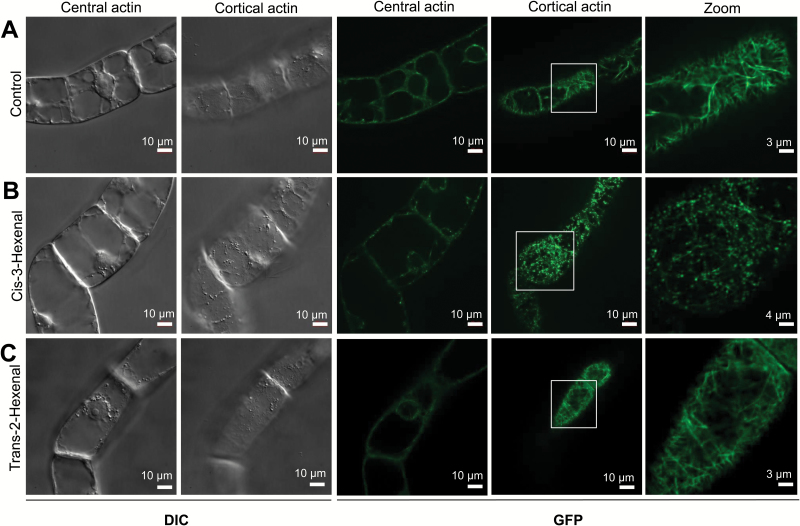
Effect of the two volatile products of VvHPL1 on the transgenic BY-2 tobacco cell line overexpressing the actin-binding domain of plant fimbrin in fusion with GFP. (A) Responses of BY-2 cells to the solvent control (triacetin). (B) Treatment with *cis*-3-hexenal and (C) treatment with *trans*-2-hexenal. Cells were collected at day 3 after subcultivation on 0.8% MS agar, and 1 µl of *cis*-3-hexenal, *trans*-2-hexenal, or the solvent triacetin/water was placed on 10 mm sterile filter paper discs in the centre of the MS agar plate and incubated for 10 min such that the compound targeted the cells exclusively through the gas phase. After incubation, cells were transferred to MS medium. For each treatment, a representative confocal section from a *z*-stack along with the differential interference contrast (DIC) image of the same cell, visualization of GFP fused with actin, and zoom-in of cortical actin are shown.

**Fig. 7.**
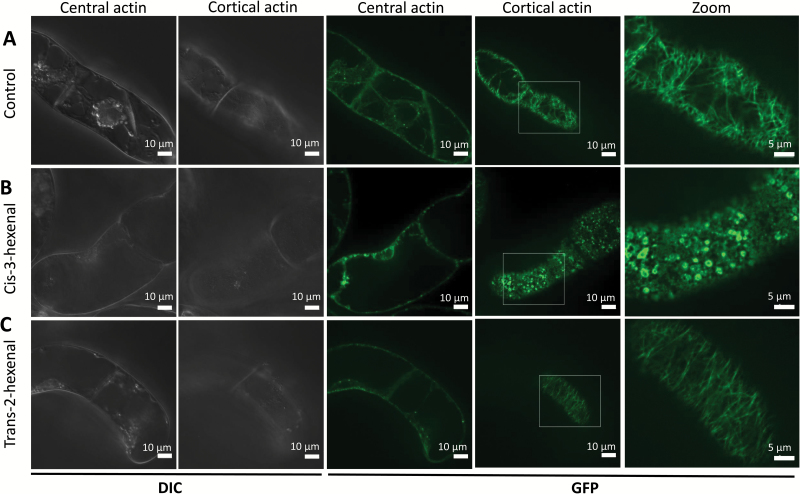
Effect of the two volatile products of VvHPL1 on transgenic cells of *Vitis vinifera* cv. ‘Chardonnay’ overexpressing the actin-binding domain of plant fimbrin in fusion with GFP. (A) Responses of grapevine cells to the solvent control (triacetin). (B) Treatment with *cis*-3-hexenal and (C) treatment with *trans*-2-hexenal. Details are given in the legend of Fig. 6.

The potential cellular effect of the two cognate VvHPL1 products, *cis*-3-hexenal and *trans*-2-hexenal, was examined. Since cell death-related immunity as elicited by harpin causes a rapid disassembly of the cortical actin filaments subtending the membrane as one of the earliest cellular hallmarks of ensuing cell death (Guan *et al.*, 2013), we investigated actin responses to these two C_6_ volatiles using a transgenic BY-2 tobacco cell line, where actin was visualized by the actin-binding domain of plant fimbrin in fusion with GFP (Sano *et al.*, 2005). In the solvent control, a rich meshwork of cortical actin filaments was observed (Fig. 6A). After treatment with *trans*-2-hexenal, we were not able to detect any significant difference from the solvent control (Fig. 6C). In contrast, treatment with *cis*-3-hexenal caused a rapid and strong disintegration of the meshwork, being already visible at the earliest time point (10 min after the start of treatment; Fig. 6B). This response of actin to *cis*-3-hexenal was followed a few minutes later by a strong increase in mortality as evident from the Evans Blue dye exclusion assay (Supplementary Fig. S5). Already 30 min after the onset of the volatile treatment, the majority of cells had died. In contrast, there was no such increase of mortality seen for treatment with *trans*-2-hexenal. Thus, there is a stereo-specific, rapid, and drastic response of cortical actin to C_6_ volatiles. We asked then whether this actin response was confined to tobacco BY-2 cells, and therefore generated a suspension cell line from grapevine (*V. vinifera* cv. ‘Chardonnay’) expressing the same marker (the actin-binding domain of plant fimbrin in fusion with GFP; Guan *et al.*, 2014). Again, neither the solvent control (Fig. 7A) nor the treatment with *trans*-2-hexenal (Fig. 7C) caused any significant effect on actin filaments, while *cis*-3-hexenal rapidly eliminated the actin meshwork (Fig. 7B). Interestingly, the fluorescent signal accumulated around the proplastids in the form of periplastidic rims. Thus, the pattern seen in tobacco BY-2 cells as a heterologous system could be reproduced in cells from grapevine itself. To obtain insight into tissue-dependent responses, we conducted a third series of experiments, where the two C_6_ volatiles were administered to the adaxial or abaxial (Supplementary Fig. S7) side of leaf discs from grapevine plants expressing the same actin marker (Guan *et al.*, 2014). We observed that actin filaments in the pavement cells of the adaxial epidermis were rapidly disrupted into punctate structures in response to *cis*-3-hexenal. In contrast to the two cell lines (tobacco BY-2 and grapevine cells), actin was also eliminated by *trans*-2-hexenal (even more thoroughly). A similar pattern was also observed in the abaxial epidermis, where actin filaments were disrupted in both pavement and guard cells in response to *cis*-3-hexenal, but also in response to *trans*-2-hexenal. Thus, while *cis*-3-hexenal causes disintegration of actin in both suspension cells and cells in a tissue context, *trans*-2-hexenal, which is not active in isolated cells, can exert an effect upon actin for cells within a tissue.

### *HPL1* accumulates in response to wounding

In response to mechanical wounding, GLVs are released in plants by the action of HPL. Therefore, the steady-state transcript level of *VvHPL1* was examined in response to wounding. As shown in Supplementary Fig. S6, mechanical wounding increased the accumulation of *HPL1* transcripts in leaves from *V. vinifera* cv. ‘Müller-Thurgau’. Transcripts had increased to ~30-fold of the initial level at 2 h after wounding and subsequently dropped slowly to ~10-fold of the initial level 6 h after wounding.

## Discussion

In the current work, we have isolated and characterized a member of the fatty acid hydroperoxide lyase family from the white grapevine (*V. vinifera* L.) cultivar ‘Müller-Thurgau’. By recombinant expression and precursor feeding experiments, we can show that this HPL1 member is a canonical member of the CYP74B family, confirming the findings on the HPL1 homologue from the red cultivar ‘Cabernet Sauvignon’ (Zhu *et al.*, 2012). We have shown that it is localized in the plastids, up-regulates defence-related cell death, and acts antagonistically with JA. We further show that one of the enzymatic products of this HPL, 3-*cis*-hexenal specifically disrupts actin filaments as a hallmark of PCD.

### VvHPL1 is a plastid-located CYP74B generating 2-hexenal

The HPL1 from *V. vinifera* cv. ‘Müller-Thurgau’ (VvHPL1) was predicted to be a member of the CYP74B clade favouring 13-hydroperoxy fatty acids and generating C_6_ volatiles. In the current work, the enzymatic activity has been confirmed using recombinant protein. VvHPL1 was indeed a true CYP74B specifically accepting 13-hydroperoxy fatty acids as substrates, giving rise to the *cis*- and the *trans*-isomers of the C_6_ volatile 2-hexenal (*cis*-3-hexenal and *trans*-2-hexenal) similar to its homologue from cv. ‘Cabernet Sauvignon’ (Zhu *et al.*, 2012). Additionally, recombinant VvHPL1 preferred 13-HPOT over 13-HPOD as substrate. This substrate preference is similar to that of the HPL homologue from tomato (Howe *et al.*, 2000), channelling the metabolic pathway towards *cis*-3-hexenal and *trans*-2-hexenal.

The oxylipin pathway is strongly compartmentalized, and this subcellular partitioning has been studied in great detail for JA biosynthesis (reviewed in Wasternack and Hause, 2013), whereby the initial steps occur in the plastid, followed by further processing in the peroxisome, and final activation in the cytoplasm. For the concurrent oxylipin branches, this compartmentalization is less clear and there has been some controversy about the localization of HPLs: whereas all three HPL versions of rice as well as the HPL of tomato were shown to be imported into plastids using an *in vitro* import assay (Froehlich *et al.*, 2001; Chehab *et al.*, 2006), a HPL of almond was reported to accumulate in lipid droplets (Mita *et al.*, 2005). Specific 9-HPL isoforms of alfalfa were reported to be located in the cytosol and in lipid droplets (Domenico et al., 2007). These different localization patterns prompted us to study the targeting of VvHPL1 in more detail. Since all previous studies had relied either on *in vitro* assays (Froehlich *et al.*, 2001; Chehab *et al.*, 2006) or on transient expression (Mita *et al.*, 2005; Domenico *et al.*, 2007), we generated stable transformants in BY-2. VvHPL1 localized in proplastids, which was expected for a canonical 13-HPL (Froehlich *et al.*, 2001; Chehab *et al.*, 2006). Therefore, we were encouraged to use this overexpressor line to address its potential cellular functions. Quantitative phenotyping with respect to viability, mitotic index, and cell length under normal physiological conditions did not reveal any difference between the overexpressor and the non-transformed wild type. This led to the question of whether the cryptic function of VvHPL1 can be rendered manifest when lipoxygenation is stimulated by exposing this transgenic line to stress conditions, thus providing the substrates for this enzyme.

### VvHPL1 functions in cell death-related stress signalling

Oxidative burst can be conveniently induced by imposing salinity stress (grapevine cells, Ismail *et al.*, 2012; BY-2 cells, Monetti *et al.*, 2014), and this will activate lipid peroxidation (Hazman *et al.*, 2015). When cells overexpressing *HPL1* were exposed to sodium stress they were significantly more sensitive, evident from exhibiting enhanced cell death. Hence salinity-induced activation of lipoxygenation provided the substrate for the ectopic HPL1, its product apparently stimulating salinity-induced cell death. At first sight, it would appear counterintuitive why an enzyme producing a stress-induced toxic compound should confer an evolutionary advantage, but it should be kept in mind that on the organismic level, elimination of unbalanced cells can help to adapt to salinity (reviewed in Shabala, 2009).

A vigorous oxidative burst can also be evoked by activation of defence through chemical elicitors. However, the timing of this oxidative burst differs between the two layers of innate immunity (Chang and Nick, 2012): flg22 induces a late oxidative burst and does not evoke a cell death response. In contrast, harpin activates a rapid oxidative burst, which is followed by cell death (grapevine cells, Chang and Nick, 2012; BY-2 cells, Guan *et al.*, 2013). We observed that the HPL1ox cells showed only a slightdly increased mortality in response to flg22, which remained below the threshold for significance and was generally hardly detectable in both cell lines. In contrast, mortality in response to harpin was strongly promoted in a dose-dependent manner in HPL1ox, consistent with a mechanism where lipid peroxidation triggered by the early oxidative burst will generate the substrate required for the ectopic HPL to act.

The ROS inducing the elevated cell death response in the overexpressor line are released in response to a signal. If a molecule acts as a signal, its release and dissipation must be regulated. In fact, ROS are employed as common signals to induce and regulate the response to different stress factors (reviewed in Baxter *et al.*, 2014). A central player for this regulated oxidative burst is the RboH in the plasma membrane (reviewed in Marino *et al.*, 2012). To address the role of this enzyme, its activity was inhibited by application of DPI. Since this inhibitor, at low concentrations, could mitigate the increased harpin-induced mortality seen in HPL1ox, it is straightforward to assume that RboH is responsible for the release of ROS, which in turn induce lipoxygenation. There is an interesting cytological aspect in this context: RboH is located in the plasma membrane, but the lipoxygenation takes place in the plastid. Thus, the signal released by RboH has to travel, which might imply that superoxide is not a direct cause of the lipoxygenation and calls for a scenario with a second messenger. The involvement of a second factor (in addition to the superoxide generated by RboH) is also indicated by the fact that DPI could reduce the harpin-induced induction of mortality by only ~50%.

Irrespective of the molecular details leading to the stress-induced lipoxygenation, our data support an explanation where the absence of a phenotype in HPL1ox under normal conditions is caused by a lack of substrate. This absence of a phenotype in the absence of stress, the correct subcellular localization of the GFP fusion, and the fact that the the product of HPL, 3-*cis*-hexenal, causes the same cellular response as seen in the overexpressor line upon activation of stress signalling, support the notion that the HPL–GFP fusion is fully functional.

If HPL acts in cell death-related signalling, activation of the concurrent pathway (jasmonate signalling) should produce an antagonistic effect. If the activation of cell death by harpin is a manifestation of a signalling pathway acting in HR-related defence, it should be quelled by exogenous jasmonate. This implication has been experimentally shown previously for BY-2 (Andi *et al.*, 2001). Likewise, if HPL acts in cell death-related signalling, jasmonate should mitigate cell death in HPL1ox and it should do so to a larger extent as compared with the wild type. In fact, we have observed that JA pre-treatment could completely eliminate the harpin-induced mortality, which means that the effect of JA was more pronounced than that of DPI. It should be noted that the relatively high concentration (100 µM) of JA required for this phenomenon is due to the low sensitivity of BY-2 cells to JA. For instance, the alkaloid pathway as metabolic readout for stress responses requires 100 µM JA in order to be efficiently activated, while lower concentrations (10 µM) induce this only very poorly (Rajabi *et al.*, 2017).

There are different possible mechanisms for this antagonistic signalling. Since the effect was seen in a line where HPL was overexpressed, transcriptional repression as the mechanism is very unlikely. The most straightforward mechanism would be competition of (ectopic) VvHPL1 and (endogenous) AOS for a common substrate. When jasmonate increases either the abundance or enzymatic activity of AOS, this might account for the observed mitigation. The transcription of AOS can be activated by jasmonates (Laudert and Weiler, 1998), and there is also regulation of AOS by jasmonate on the post-translational level (Miersch and Wasternack, 2000; Scholz *et al.*, 2015). However, the finding that in the HPL overexpressor there is more and not less JA-Ile speaks against substrate competition as the explanation. This phenomenon must be linked with a feedback regulation of JA-Ile signalling upon JA synthesis or catabolism.

Our findings lead to the conclusion that HPL acts in a signalling pathway that functions in signal-dependent (programmed) cell death. This pathway is regulated in a dynamic way by the availability of substrate triggered by signal-dependent oxidative burst. If this model holds true, the enzymatic products generated by HPL should be able to activate PCD.

### Is the product of VvHPL1 activating cell death signalling?

The biological effect of C_6_ volatiles could be a chronic or acute toxicity; for instance, aphid fecundity was doubled on potato plants that had been genetically engineered for reduced HPL activity, which was explained by a general toxicity of GLVs (Vancanneyt *et al.*, 2001). Furthermore, the effect of short-chained aldehydes as ‘volatile phytoalexins’ (Zeringue and McCormick, 1989) is explained in terms of unspecific cytotoxicity. Alternatively, C_6_ volatiles might act as specific signals. The regulatory specificities discussed above such as the link with signal-dependent oxidative burst and the antagonism with jasmonate signalling support a scenario where the products of HPL act as cellular signals rather than as toxic executors of cell death. Whether a molecule is a signal mainly depends on the presence of a receptor. When we tested the cellular effect of the enzymatic products of VvHPL1 on a BY-2 cell line expressing a GFP–actin marker, we observed a rapid disintegration of cortical actin only for *cis*-3-hexenal (not *trans*-2-hexenal), a hallmark for PCD (Smertenko and Franklin-Tong, 2011). This isomer specificity could be confirmed in suspension cells expressing the same GFP-tagged actin marker used as a homologous system. Interestingly, in a tissue context, both isomers show activity against actin. Why *trans*-2-hexenal is more active in leaves as compared with suspension cells is currently being addressed in further experiments; it might be caused by a different stability of actin filaments in differentiated cells or by a different competence for the signal.

### Outlook

The isomer specificity of the actin response indicates that this response is likely to be activated by a specific binding site. Whether this binding site is linked with activation of a plant-based signal chain that triggers PCD will be addressed by future work dedicated to understanding the biological function and the molecular and cellular events underlying this response. To obtain insights into the function of HPL1 in grapevine, the transcriptional regulation of VvHPL1 in stress-induced adaptive necrosis (e.g. in the context of salt stress or in response to pathogens) will be investigated.

## Supplementary data

Supplementary data are available at *JXB* online.

Fig. S1. Molecular phylogeny constructed by the Neighbor–Joining algorithm on selected members of the CYP74 family.

Fig. S2. Alignment of the HPL isolated from *Vitis vinifera* cv. ‘Müller-Thurgau’ along with the two published HPL sequences VvHLP1 and VvHLP2 (Zhu *et al.*, 2012), and representative members of CYP74A (AOS from *Arabidopsis thaliana*, UniProt accession Q96242, predicted AOS from *Vitis vinifera*, UniProt accession F6H025), CYP74B (HPL from *Arabidopsis thaliana*, UniProt accession Q96242), CYP74C (HPL from *Medicago truncatulata*, UniProt accession Q7X9B3), and CYP74D (DES from *Solanum tuberosum*, UniProt accession Q9AVQ1.

Fig. S3. Modulation of harpin-induced mortality by diphenylene iodonium (DPI) in tobacco BY-2 cells overexpressing the HPL isolated from *Vitis vinifera* cv. ‘Müller-Thurgau’ in fusion with GFP at the C-terminus.

Fig. S4. Quantification of jasmonoyl-isoleucine (JA-Ile) in tobacco BY-2 cells overexpressing the HPL isolated from *Vitis vinifera* cv. ‘Müller-Thurgau’ in fusion with GFP at the C-terminus.

Fig. S5. Quantification of mortality measured by the Evans Blue dye exclusion test after treatment with either solvent (triacetin/water) or the two volatile products of VvHPL1 on BY-2 tobacco cells.

Fig. S6. Expression of *VvHPL1* in response to mechanical wounding.

Fig. S7. Effect of the two volatile products of VvHPL1 on the adaxial (A) or abaxial (B) side of leaf discs from grapevine plants expressing the actin-binding domain of plant fimbrin in fusion with GFP.

Table S1. Primers used for real-time qPCR analysis.

Table S2. Survey on substrate preference of recombinant VvHPL1 tested with different fatty acid hydroperoxides and identified products.

Table S3. Primers used for Gateway^®^ cloning

Supplementary Figures and Tables

## Abbreviations.

AOS: allene oxide synthase
BY-2: tobacco *Nicotiana tabacum* L. cv. bright yellow 2
CYP: cytochrome P_450_
DPI: diphenyleneiodonium
ETI: effector-triggered immunity
GFP: green fluorescent protein
GLV: green leaf volatile
HPL: hydroperoxide lyase
HPOD: hydroperoxy octadecadienoic acid
HPOT: hydroperoxy octadecatrienoic acid
HR: hypersensitive response
JA: jasmonic acid
JA-Ile: (+)-7-iso-jasmonoyl-l-isoleucine
PAMP: pathogen-associated molecular pattern
PCD: programmed cell death
PTI: PAMP-triggered immunity
ROS: reactive oxygen species
SBSE: stir bar sorptive extraction
WT: wild type.
