# Supplementary material for: Grapevine fatty acid hydroperoxide lyase generates actin-disrupting volatiles and promotes defence-related cell death
Source: J Exp Bot. 2018 Apr 5;69(12):2883–96. doi: 10.1093/jxb/ery133 (PMC5972561; doi:10.1093/jxb/ery133)
Supplement: Supplementary Figures and Tables [file ery133_suppl_supplementary_figures_tables.pdf]

Supplementary Figures

Supplementary Figure S1: Molecular phylogeny constructed by the neighbor-joining algorithm on selected members of the CYP74 family. The position of the HPL sequence from 'Müller-Thurgau' is indicated by a red circle. Green circles indicate proteins, where the respective enzymatic activity has been demonstrated experimentally, blue circles indicate proteins, where the respective enzymatic activity has been inferred from the presence of the respective specificity signatures in the sequence. Values next to the branches represent the percentage of replicate trees in which the associated taxa clustered together in the bootstrap test (based on 500 replicates). The tree is drawn to scale, with branch lengths in the same units as those of the evolutionary distances used to infer the phylogenetic tree. The evolutionary distances were computed using the Poisson correction method and are in the units of the number of amino acid substitutions per site. The Swissprot accessions of the shown sequences are: CYP74A (AOS): *Na* AOS *Nicotiana attenuata* Q8W4X8, verified experimentally (Ziegler *et al.,* 2001); *Nt* AOS *Nicotiana tabacum* L8B431, predicted from signature; *St* AOS *Solanum tuberosum* Q8H1X6, predicted from signature, localization verified experimentally (Farmaki *et al.,* 2006); *Sl* AOS *Solanum lycopersicum* Q9M464, predicted from signature; *Gm* AOS *Glycine max* A0MAV6, verified experimentally (Wu *et al.,* 2008); *Vv* AOS *Vitis vinifera* F6H025, predicted from signature; *At* AOS *Arabidopsis thaliana* Q96242, verified experimentally (Lee *et al.,* 2008); *Os* AOS *Oryza sativa* Q7XYS3, verified experimentally (HA *et al.,* 2002); *Zm* AOS *Zea mays* Q6RW10, predicted from signature. CYP74B (13-HPL): *Sm* 13-HPL 1 *Selaginella moellendorffi* D8RA26, predicted from signature; *Sm*13-HPL 2 *Selaginella moellendorffi* D8QZ31, predicted from signature; *Os* 13-HPL *Oryza sativa* Q7X9C2, predicted from signature; *At* 13-HPL *Arabidopsis thaliana* Q9ZSY9, verified experimentally (Bate *et al.,* 1998); *Na* 13-HPL *Nicotiana attenuata* Q93YF8, predicted from signature; *Nt* 13-HPL *Nicotiana tabacum* Q45KF8, predicted from signature; *Sl* 13-HPL *Solanum lycopersicum* Q9ARH8, verified experimentally (Matsui *et al.,* 2000); *Lu* 13-HPL *Linumusitatissimum* E3VWA8, verified experimentally (Gogolev *et al.,* 2012); *Mt* 13-HPL *Medicago truncatulata* Q4ZGM9, predicted from signature; VvCabSauv 13-HPL *Vitisvinifera*cv. 'Cabernet Sauvignon'HPL1 E5FXI7, verified experimentally (Zhu *et al.,* 2012); Vv MTh 13-HPL *Vitis vinifera* cv. 'Müller-Thurgau' this work; KX379687. VvPinNo 13-HPL. Ancient HPL: *Wn* HPL *Wollemia nobilis* A0A0C9QM15, predicted from signature, no plastid localization sequence; *Ps* HPL1 *Picea sitchensis* A9NX03, predicted from signature, no plastid localisation sequence; *Ps* HPL2 *Picea sitchensis* B8LKD1, predicted from signature, no plastid localisation sequence; *Ps* HPL3 *Picea sitchensis* B8LK69, predicted from signature, no plastid localisation sequence; *Sm* CYP74 *Selaginella moellendorfii* D8T6U4, predicted from signature, plastid localisation sequence verified; *Pp* CYP74 1 *Physcomitrella patens* A9SNA2, predicted from signature, plastid localisation sequence verified; *Pp* CYP74 1 *Physcomitrella patens* A9S014, predicted from signature, plastid localisation sequence verified. CYP74C (9-13-HPL): *Mt* 9-13 HPL *Medicago truncatulata* Q7X9B3, predicted from signature; *Cm* 9-13 HPL *Cucumis melo* Q93XR3, verified experimentally (Tijet *et al.,* 2001); *Pd* 9-13 *Prunus dulcis* Q7XB42, verified experimentally (Mita *et al.,* 2005); *VvCabSauv* 9-13 HPL *Vitis vinifera*

cv. 'Cabernet Sauvignon' HPL2 E5FXI8, verified experimentally (Zhu *et al.,* 2012); *VvPinNo* 9-13 HPL *Vitis vinifera*cv. 'Pinot Noir' F6HQI4, predicted from signature. CYP74D (DES): *Sl* DES *Solanum lycopersicum* Q9FPM6, verified experimentally (Itoh and Howe 2001); *St* DES *Solanum tuberosum* Q9AVQ1, verified experimentally (Stumpe *et al.,* 2001); *Ca* DES *Capsicum annuum* Q0PHS9, predicted from signature; Nt DES *Nicotiana tabacum* Q8W2N5, verified experimentally (Fammartino *et al.,* 2007). There are, of course, additional sequences such as *St* AOS *Solanum tuberosum* CAI30876 (Stumpe *et al.,* 2006), or *Mt*HPL *Medicago truncatulata* CAC86898 that verified experimentally (Stumpe *et al.,* 2005).

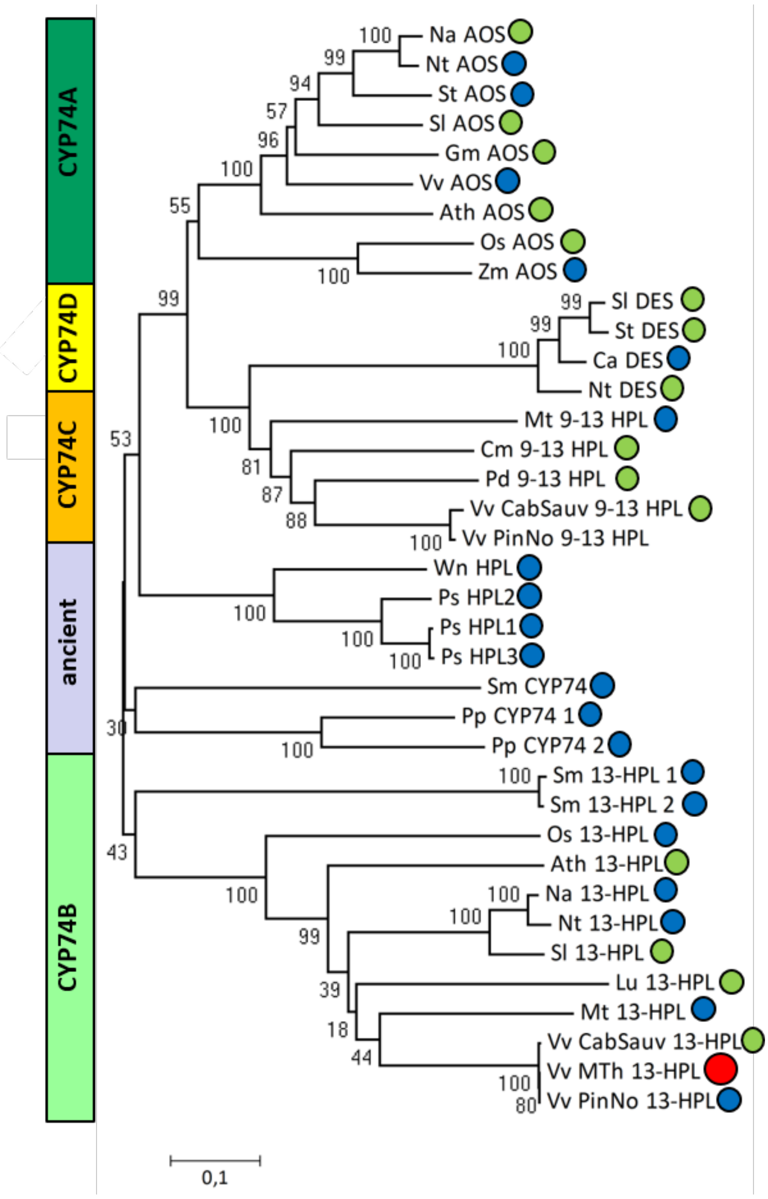

Supplementary Figure S2: Alignment of the HPL isolated from *Vitis vinifera* cv. 'Müller-Thurgau' along with the two published HPL sequences VvHLP1 and VvHLP2 (Zhu *et al.,* 2012), and representative members of CYP74A (AOS from *Arabidopsis thaliana*, UniProt accession Q96242, predicted AOS from *Vitis vinifera*, UniProt accession F6H025), CYP74B (HPL from *Arabidopsis thaliana*, UniProt accession Q96242), CYP74C (HPL from *Medicago truncatulata*, UniProt accession Q7X9B3), and CYP74D (DES from *Solanum tuberosum*, UniProt accession Q9AVQ1. The following features are indicated in the sequences:

- Interaction sites with haem propionate marked as
- Highly conserved cysteine residue (iron binding) at C-terminus marked as
- The CYP74 characteristic signature FXXG-9 gaps – XXXCXG
- Specificity defining F in AOS that mutated to L converts AOS to HPL (Lee *et al.,* 2008)
- Specificity defining L in AOS that is converted to V (Lee *et al.,* 2008)
- Specificity defining S in AOS that is converted to A (Lee *et al.,* 2008)
- Specificity defining NAFGG in 74C (substrate binding pocket, Toporkova *et al.,* 2013)
- Specificity defining WV in 74C (substrate binding pocket, Hughes *et al.,* 2009)
- Substrate recognition site (I-helix) is indicated by ____ (corresponds to oxygen binding in canonical cytochrome-P450, Toporkova *et al.,* 2013)
- ERR triade domain (interaction with the I-helix and important for substrate specificity) with a 74B specific C and a DES specific F (mutation of this V to F will generate AOS activity, Toporkova *et al.,* 2013)

CLUSTAL W (1.83) multiple sequence alignment
Parameters
-matrix=blosum -gapopen=10 -gapext=0.05 -endgaps=10 -gapdist=0.05

```
Predicted plastidic transit peptides    ______________________________________________

HPL_MTh                               ----------------------------MLSSTVMSVSPGVPTPSSLT
vHPL1_Cabernet_Sauvignon            ----------------------------MLSSTVMSVSPGVPTPSSLT--
VvHPL2_Cabernet_Sauvignon           ----------------------------------MSSSSSSLPLNFVN--
AOS_Vitis_F6H025_predicted          MASPSLTFPSLQLQFPTHTKSSKPSNHKLIVRPIFASVSEKPSVPVSQ--
Arabidopsis_C74A_AOS_Q96242         MASISTPFP---ISLHPKTVRSKPLKFRVLTRPIKASGSETPDLTVAT--
Arabidopsis_C74B2_HPL_Q9ZSY9        ----------------------------MLLRTMAATSPRPPPSTSLTSQ
Medicago_C74C_HPL_ Q7X9B3           -------------------------------------------MASSS--
Solanum_tuberosum__C74D_DES_Q9AVQ1  ---------------------------------------------MSS--



                                                                        ____________

HPL_MTh                             -PPSPPSSSPVRAIPGSYGWPVLGPIADRLDYFWFQGPETFFRKRIDKYK
VvHPL1_Cabernet_Sauvignon           -PPSPPSSSPVRAIPGSYGWPVLGPIADRLDYFWFQGPETFFRKRIDKYK
VvHPL2_Cabernet_Sauvignon           --SSSSSKLPLRSIPGDCGSPFFGPIKDRFDYFYNEGRDPFFRTRMQKYQ
AOS_Vitis_F6H025_predicted          --SQVTPPGPIRKIPGDYGLPFIGPIKDRLDYFYNQGREEFFRSRAQKHQ
Arabidopsis_C74A_AOS_Q96242         --RTGSKDLPIRNIPGNYGLPIVGPIKDRWDYFYDQGAEEFFKSRIRKYN
Arabidopsis_C74B2_HPL_Q9ZSY9        QPPSPPSQLPLRTMPGSYGWPLVGPLSDRLDYFWFQGPDKFFRTRAEKYK
Medicago_C74C_HPL_ Q7X9B3           --ETSSTNLPLKPIPGSYGLPIIGPLHDRHDYFYNQGRDKYFQTRIEKYN
Solanum_tuberosum__C74D_DES_Q9AVQ1  --YSELSNLPIREIPGDYGFPIISAIKDRYDYFYNQGEDAWFHNKAEKYK
                                             *:: :**. * *....: ** ***: :* : :*:.:  *::

HPL_MTh                             STVFRTNVPPSFPFFVGVNPNVIAVLDCKSFSFLFDMDVVEKKNVLVGDF
VvHPL1_Cabernet_Sauvignon           STVFRTNVPPSFPFFVGVNPNVIAVLDCKSFSFLFDMDVVEKKNVLVGDF
VvHPL2_Cabernet_Sauvignon           STVFRANMPPGP--FMALNPNVVVLLDAISFPILFDTSRIEKRNVLDGTY
AOS_Vitis_F6H025_predicted          STVFRSNMPPGP--FISSNSKVIVLLDGKSFPVLFDVSKVEKKDVFTGTF
Arabidopsis_C74A_AOS_Q96242         STVYRVNMPPGA--FIAENPQVVALLDGKSFPVLFDVDKVEKKDLFTGTY
Arabidopsis_C74B2_HPL_Q9ZSY9        STVFRTNIPPTFPFFGNVNPNIVAVLDVKSFSHLFDMDLVDKRDVLIGDF
Medicago_C74C_HPL_ Q7X9B3           STVLKLNMPPGG--FIAPDPKVIALLDGASFPILFDNAKVEKRDVLDGTF
Solanum_tuberosum__C74D_DES_Q9AVQ1  STVVKINMAPGP--FTSNDYKLVAFLDANSFVCMFDNSLIDKTDTLGGTF
                                    *** : *:.*    *   : :::..**  **  :**   ::* : : * :

HPL_MTh                             MPSVKYTGDIRVCAYLDTAETQHARVKSFAMDILKRSSSIWASEVVASLD
VvHPL1_Cabernet_Sauvignon           MPSVKYTGDIRVCAYLDTAETQHARVKSFAMDILKRSSSIWASEVVASLD
VvHPL2_Cabernet_Sauvignon           MPSTAFTGGYRVCAYLDPSEPNHALLKRLFTSSLAARHHNFIPVFRSCLT
AOS_Vitis_F6H025_predicted          MPSTEFTGGFRVLSYLDPSEPDHTKLKRLLFFLLQSSRDRVIPEFHSCFS
Arabidopsis_C74A_AOS_Q96242         MPSTELTGGYRILSYLDPSEPKHEKLKNLLFFLLKSSRNRIFPEFQATYS
Arabidopsis_C74B2_HPL_Q9ZSY9        RPSLGFYGGVCVGVNLDTTEPKHAKIKGFAMETLKRSSKVWLQELRSNLN
Medicago_C74C_HPL_ Q7X9B3           MPSTDFFGGYRTCAFQDTAEPSHSLLKRFIFHILSSKHDTFIPLFQTNLT
Solanum_tuberosum__C74D_DES_Q9AVQ1  KPGKEYYSGYRPVAFIDTKDPNHAALKGYILSAFAKRHNLFIPLFRNSLS
                                     *.    ..       *. :..*  :*      :          .

HPL_MTh                             TMWDTIDAGVA-KSNSASYIKPLQRFIFHFLTKCLVGADPAVSPEIAESG
VvHPL1_Cabernet_Sauvignon           TMWDTIDAGVA-KSNSASYIKPLQRFIFHFLTKCLVGADPAVSPEIAESG
VvHPL2_Cabernet_Sauvignon           ELFTTLEDDVS-RKGKADFNGISDNMSFNFVFKLFCDKH-PSETKLGSNG
AOS_Vitis_F6H025_predicted          ELSETLESELAAKG-KASFADPNDQASFNFLARALYGTK-PADTKLGTDG
Arabidopsis_C74A_AOS_Q96242         ELFDSLEKELSLKG-KADFGGSSDGTAFNFLARAFYGTN-PADTKLKADA
Arabidopsis_C74B2_HPL_Q9ZSY9        IFWGTIESEIS-KNGAASYIFPLQRCIFSFLCASLAGVDASVSPDIAENG
Medicago_C74C_HPL_ Q7X9B3           EHFTDLEKELAGKHQKASFNTSIGGITFNFLFKLITDKN-PSETKIGDSG
Solanum_tuberosum__C74D_DES_Q9AVQ1  DHLFNNLEKQVTEQGKSDFNALLPTMTFNFIFRLLCDQTNPSDTVLGAQG
                                                .   :.:        * *:   : .   . .. :  ..

HPL_MTh                             YVMLDKWVFLQLLPTISVN--FL-QPLEEIFLHSFAYPFFLVKGDYRKLY
VvHPL1_Cabernet_Sauvignon           YVMLDKWVFLQLLPTISVN--FL-QPLEEIFLHSFAYPFFLVKGDYRKLY
VvHPL2_Cabernet_Sauvignon           PNLVTKWLFLQLAPFITLGLSMLPNVVEDLLLHTFPLPSLFVKSDYKKLY
AOS_Vitis_F6H025_predicted          PGLITTWVVFQLSPILTLG---LPKFIEEPLIHTFPLPAFLAKSSYQKLY
Arabidopsis_C74A_AOS_Q96242         PGLITKWVLFNLHPLLSIG---LPRVIEEPLIHTFSLPPALVKSDYQRLY
Arabidopsis_C74B2_HPL_Q9ZSY9        WKTINTWLALQVIPTAKLG--VVPQPLEEILLHTWPYPSLLIAGNYKKLY
Medicago_C74C_HPL_ Q7X9B3           PTLVQTWLAAQLAPLATAGLPKIFNYLEDVLIRTIPIPAWTVKSSYNKLY
Solanum_tuberosum__C74D_DES_Q9AVQ1  PEHLRKWLFPQLIPSLSA--KKLPNIIEDTLFHNFLIPFGFIKSDYNKLV
                                       : .*:  :: *  .     : . :*: :::.   *     ..*.:*
```

I-Helix

```
                                                     _________________________________

HPL_MTh                             DFVEQHGQAVLQRGETEFNLSKEETIHNLLFVLGFNAFGGFTIFFPSLLS
VvHPL1_Cabernet_Sauvignon           DFVEQHGQAVLQRGETEFNLSKEETTHNLLFVLGFNAFGGFTIFFPSLLS
VvHPL2_Cabernet_Sauvignon           HAFYASASSILDEAE-SMGIKRDEACHNLVFLAGFNACGGMKTLFPALIK
AOS_Vitis_F6H025_predicted          DFFYDASTHVLDEGE-KMGISREEACHNLLFATCFNSFGGMKIIFPTILK
Arabidopsis_C74A_AOS_Q96242         EFFLESAGEILVEAD-KLGISREEATHNLLFATCFNTWGGMKILFPNMVK
Arabidopsis_C74B2_HPL_Q9ZSY9        NFIDENAGDCLRLGQEEFRLTRDEAIQNLLFVLGFNAYGGFSVFLPSLIG
Medicago_C74C_HPL_ Q7X9B3           EGLMEAGTTVLDEAE-K-GIKREEACHNLVFTLGFNAFGGLTNQFPILIK
Solanum_tuberosum__C74D_DES_Q9AVQ1  DAFSKSAVSILDEAE-K-GIKREEAVQNILFLVGINMFAGLNAFSPHLFR
                                    . .   .   *  .: .: :.::*: :*::*   :*  .*:.   * :.
```

ERR-triade

```
                                    ______                                      ______

HPL_MTh                             ALSGKPE-LQAKLREEVRSKIKP-GTNLTFESVKDLELVHSVVYETLRLN
VvHPL1_Cabernet_Sauvignon           ALSGKPE-LQAKLREEVRSKIKP-GTNLTFESVKDLELVHSVVYETLRLN
VvHPL2_Cabernet_Sauvignon           WVGLAGEKLHRQLADEIRSIVKAEG-GVTFAALDKMALTKSVVYEALRIE
AOS_Vitis_F6H025_predicted          WVGRGGVKLHTQLAQEIRSVVKSNGGKVTMASMEQMPLMKSTVYEAFRIE
Arabidopsis_C74A_AOS_Q96242         RIGRAGHQVHNRLAEEIRSVIKSNGGELTMGAIEKMELTKSVVYECLRFE
Arabidopsis_C74B2_HPL_Q9ZSY9        RITGDNSGLQERIRTEVRRVCGS-GSDLNFKTVNEMELVKSVVYETLRFN
Medicago_C74C_HPL_ Q7X9B3           WVGLAGADLHKKLADEIRAIVREEG-GVNLYALDKMTLTKSTVYEALRIE
Solanum_tuberosum__C74D_DES_Q9AVQ1  FVGEAGASLHTQLAKEIRTVIKEEGGAITLSAINKMSLVKSVVYETLRLR
                                     :      :: ::  *:*      *  :.: ::..: * :*.*** :*:.
```

ERR-triade

```
                                    __________________________________________________

HPL_MTh                             PPVPLQYARARKDFQLSSHDSVFEIKKGDLLCGFQKVAMTDPKIFDDPET
VvHPL1_Cabernet_Sauvignon           PPVPLQYARARKDFQLSSHDSVFEIKKGDLLCGFQKVAMTDPKIFDDPET
VvHPL2_Cabernet_Sauvignon           PPVPFQYGKAKEDMVIHSHDAAFVIKKGEMIFGYQPFATKDPKVFDNPEE
AOS_Vitis_F6H025_predicted          PPVALQYGKAKQDLVIESHDSVFEVKEGEMLFGYQPFATKDPKIFERSEE
Arabidopsis_C74A_AOS_Q96242         PPVTAQYGRAKKDLVIESHDAAFKVKAGEMLYGYQPLATRDPKIFDRADE
Arabidopsis_C74B2_HPL_Q9ZSY9        PPVPLQFARARKDFQISSHDAVFEVKKGELLCGYQPLVMRDANVFDEPEE
Medicago_C74C_HPL_ Q7X9B3           PAVPYQYAKAREDLVVQSHDASFEIKKGEMIFGYQPFATKDAKIFDKPED
Solanum_tuberosum__C74D_DES_Q9AVQ1  PPVPLQYGKAKKDFMVQSHDASYKINKGQFVVGYQPMASRDPKIFANPDE
                                    *.*. *:.:*::*: : ***: : :: *::: *:* ..  *.::*  .:

                                                                                ______

HPL_MTh                             FVPDRFTKEKGRELLNYLFWSNGPQTGSPSDRNKQCAAKDYVTMTAVLFV
VvHPL1_Cabernet_Sauvignon           FVPDRFTKEKGRELLNYLFWSNGPQTGSPSDRNKQCAAKDYVTMTAVLFV
VvHPL2_Cabernet_Sauvignon           FVAHRFMGD-GEKLLEYVYWSNGRESDDATVENKQCPGKDLVVLLSRVML
AOS_Vitis_F6H025_predicted          FVPDRFVGE-GEKLLKHVLWSNGPETENPTLGNKQCAGKDFVVLAARLFV
Arabidopsis_C74A_AOS_Q96242         FVPERFVGEEGEKLLRHVLWSNGPETETPTVGNKQCAGKDFVVLVARLFV
Arabidopsis_C74B2_HPL_Q9ZSY9        FKPDRYVGETGSELLNYLYWSNGPQTGTPSASNKQCAAKDIVTLTASLLV
Medicago_C74C_HPL_ Q7X9B3           FIAERFIGD-GEKLLKHVFWSNGRETDEATPDNKICPAKNLVVLLCRLYL
Solanum_tuberosum__C74D_DES_Q9AVQ1  FVPDRFMND-GEKMLKHVLWSNGRETENPAPDNKQCPGKDLVHLLGRLIL
                                    * ..*:  : * ::*.:: **** ::  .:  ** *..*: * :   : :
                                                                   FXXG         XXXCXG

HPL_MTh                                    THMFQRYDSVTASGSSITAVEKAN-------------------
VvHPL1_Cabernet_Sauvignon                  THMFQRYDSVTASGSSITAVEKAN-------------------
VvHPL2_Cabernet_Sauvignon                  VEFFLHYDTFDIEYGTLLLGSSVTFKSLTKQPTFDHKSIKHVS
AOS_Vitis_F6H025_predicted                 VELFLRYDSFDIEVGTSLLGSAINLTSLKRASF----------
Arabidopsis_C74A_AOS_Q96242                IEIFRRYDSFDIEVGTSPLGSSVNFSSLRKASF----------
Arabidopsis_C74B2_HPL_Q9ZSY9               ADLFLRYDTITGDSGSIKAVVKAK-------------------
Medicago_C74C_HPL_ Q7X9B3                  VEFFLNYDTFTFDFKPSVLGPTITIKSLVKASSTV--------
Solanum_tuberosum__C74D_DES_Q9AVQ1         VEFFMRYDTFTVEITPLFRAPNVAFKTLTKASK----------
                                            .:* .**:.  .  .
```

Supplementary Figure S3: Modulation of harpin-induced mortality by Diphenylene Iodonium (DPI) in tobacco BY-2 overexpressing the HPL isolated from *Vitis vinifera* cv. 'Müller-Thurgau' in fusion with GFP at the C-terminus. Effect of the NADPH-oxidase inhibitor DPI (0.2 µM) on cell death induced by harpin (27 $\mu g \cdot ml^{-1}$) in the non-transformed BY-2 cells (white bars) versus cells overexpressing HPL (black bars). Mortality was scored after 24 h of treatment. Treatment was started 30 minutes after subcultivation. Values represent mean and standard errors from a population of 1500 cells per measurement from at least three independent experimental series. Brackets indicate differences that are significant at *P* = 0.05 (*) or *P* = 0.01 (**), Student's *t*-test.

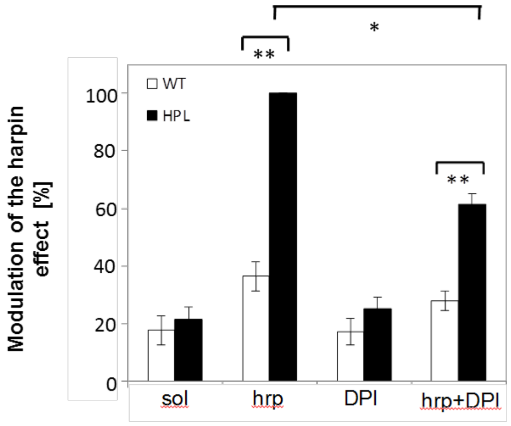

Supplementary Figure S4: Quantification of Jasmonoyl-Isoleucine (JA-Ile) in tobacco BY-2 overexpressing the HPL isolated from *Vitis vinifera* cv. 'Müller-Thurgau' in fusion with GFP at the C-terminus. Effect of the harpin (27 µg·ml^{-1}) in the non-transformed BY-2 cells (white bars) versus cells overexpressing HPL (black bars) on JA-Ile level. The JA-Ile levels were quantified after 30 minutes and 3 hours of treatment along with control samples without harpin. Values represent mean and standard errors per measurement from at least three independent experimental series. Brackets indicate differences that are significant at $P = 0.05$ (*), Student's *t*-test.

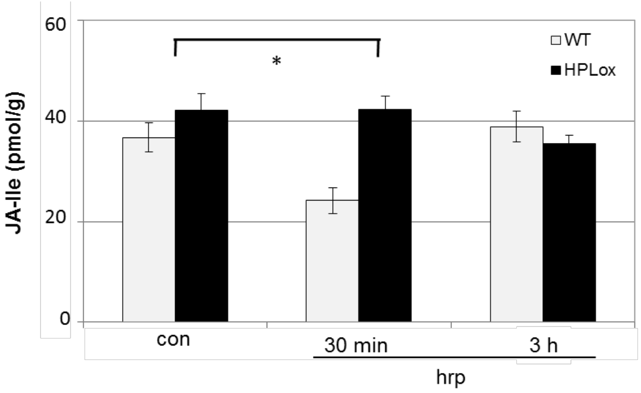

Supplementary Figure S5: Quantification of mortality measured by the Evans Blue dye exclusion test after treatment with either solvent (triacetin/water), or the two volatile products of VvHPL1 on BY-2 tobacco cells. Cells were collected at day 3 after subcultivation on 0.8% MS-agar, and 1 µl of cis-3-hexenal, trans-2-hexenal, or the solvent triacetin/water were placed on 10 mm sterile filter paper discs in the centre of the MS-agar plate and incubated for 10 minutes such that the compound targeted the cells exclusively through the gas phase. After incubation, cells were transferred to MS-medium and scored at the indicated time points (measured from the onset of the volatile treatment). Data represent mean values and standard errors from four independent experimental series comprising a population of 500 scored cells per experiment. Brackets indicate differences that are significant at *P* = 0.05 (*) or *P* = 0.01 (**), Student's *t*-test.

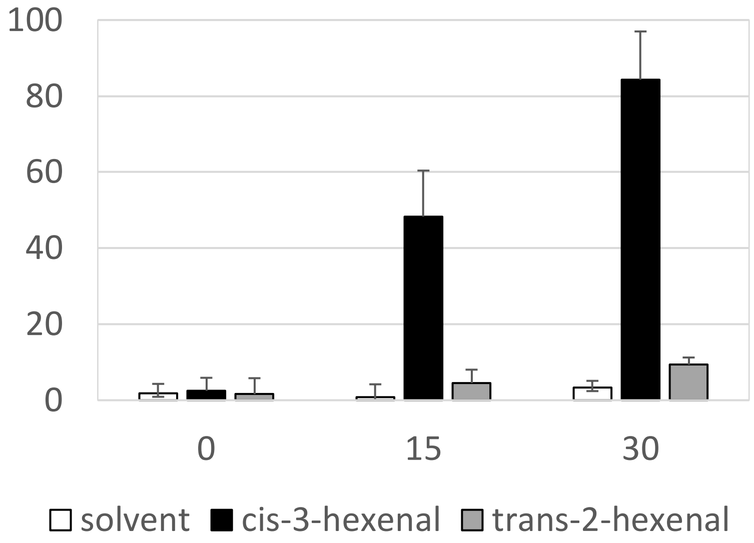

Supplementary Figure S6: Expression of *VvHPL1* in response to mechanical wounding. Quantification of *HPL1* transcripts in *Vitis vinifera* cv. 'Müller-Thurgau' leaf by quantitative real-time PCR normalized to the expression of ubiquitin conjugating enzyme (UBC). Data represent mean values from three independent experimental series, error bars represent standard errors. Brackets indicate differences that are significant at *P* <0.01 (**), Student's *t*-test.

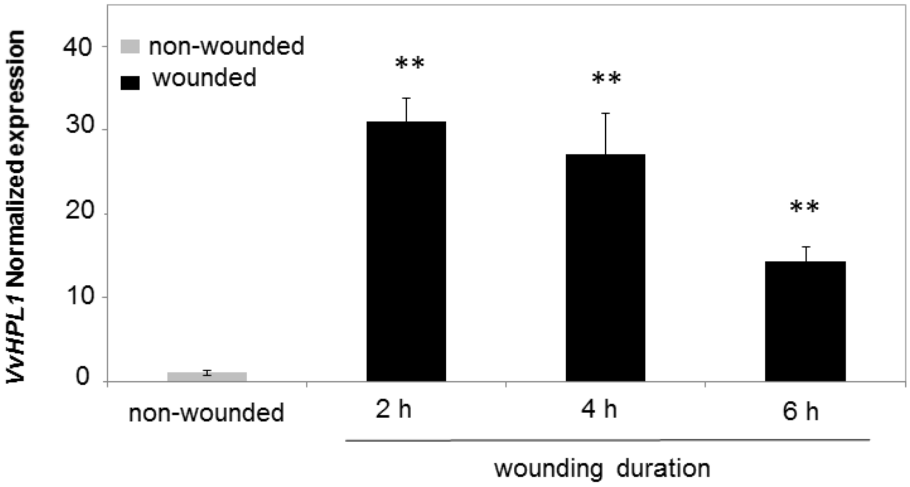

Supplementary Figure S7: Effect of the two volatile products of VvHPL1 on the adaxial (A) or abaxial (B) side of leaf discs from grapevine plants expressing actin-binding domain of plant fimbrin in fusion with GFP. **A** Responses of the cells in the adaxial and abaxial epidermis to the solvent control (triacetin). **B** Treatment with Cis-3-hexenal, and **C** Treatment with trans-2-hexenal.

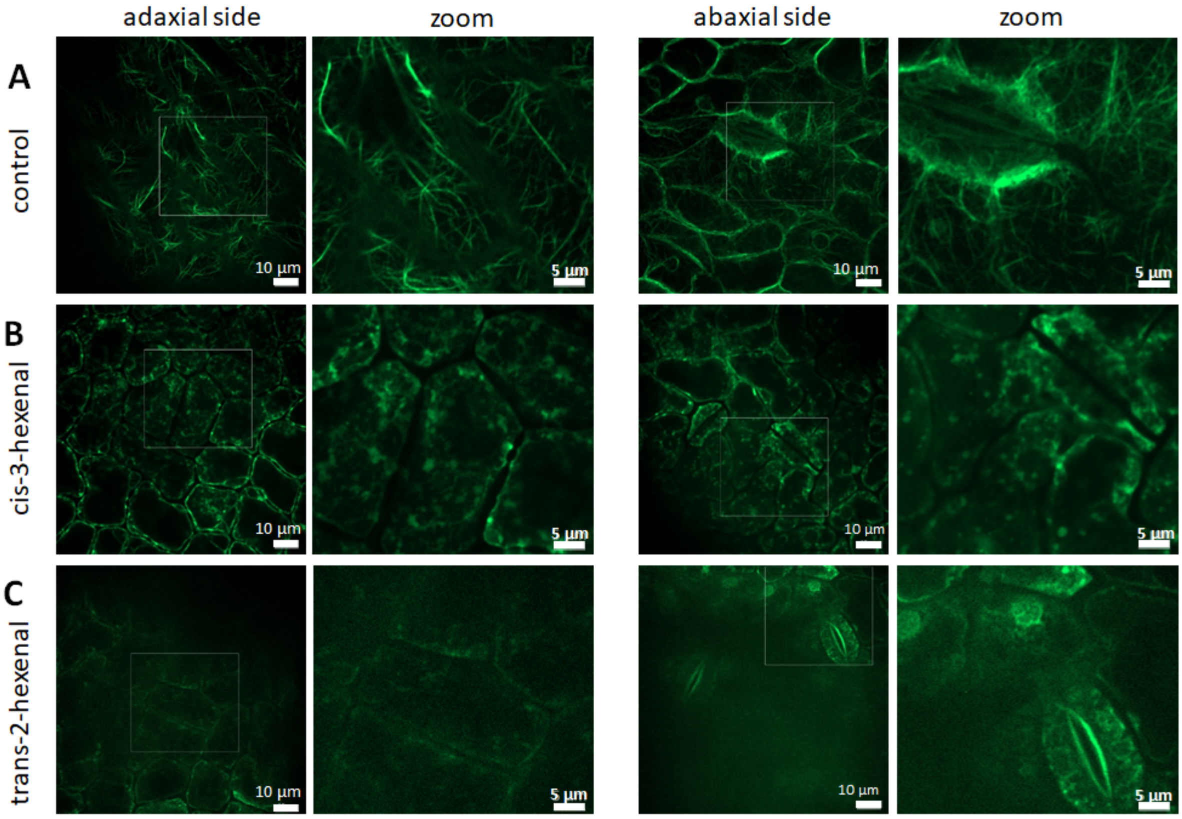

Supplementary Tables

Supplementary Table 1: Primers used for real-time qPCR analysis

Primers	Sequences (5'- 3')
HPL Fw	AGCCGGGAACAAATCTAACC
HPL Re	TTTCTGGCTCGAGCGTATTG
UBC Fw	GAGGGTCGTCAGGATTTGGA
UBC Re	GCCCTGCACTTACCATCTTTAAG

Supplementary Table 2: Survey about substrate preference of recombinant VvHPL1 tested with different fatty acid hydroperoxides and identified products.

	Products							
Substrate	n-hexanal	trans-2-hexenal	cis-3-hexenal	trans-2-hexenol	cis-3-hexenol	nonanal	2-nonenal	(*Z*,*Z*)-3,6-nonadienal
13-HPOT	-	+	+	-	-	-	-	-
13-HPOD	+	-	-	-	-	-	-	-
9-HPOD	-	-	-	-	-	-	-	-
9-HPOT	-	-	-	-	-	-	-	-

Supplementary Table 3: Primers used for Gateway® cloning

Primers	Sequences (5'- 3')
**VvHPL1 Fw**	GGGG ACA AGT TTG TAC AAA AAA GCA GGC TTCATG TTG TCT TCC ACG GTC ATG
**VvHPL1 Re N-terminal**	GGGG AC CAC TTT GTA CAA GAA AGC TGG GTC TCA GTT AGC TTT CTC AAC GGC GGTG
**VvHPL1 Re C-terminal**	GGGG AC CAC TTT GTA CAA GAA AGC TGG GTC GTT AGC TTT CTC AACGGC GGTG
